# Pre-natal exposures and breast tissue composition: findings from a British pre-birth cohort of young women and a systematic review

**DOI:** 10.1186/s13058-016-0751-z

**Published:** 2016-10-12

**Authors:** Rachel Denholm, Bianca De Stavola, John H. Hipwell, Simon J. Doran, Marta C. Busana, Amanda Eng, Mona Jeffreys, Martin O. Leach, David Hawkes, Isabel dos Santos Silva

**Affiliations:** 1 Department of Non-Communicable Disease Epidemiology, London School of Hygiene & Tropical Medicine, London, UK; 2 Department of Medical Statistics, London School of Hygiene & Tropical Medicine, London, UK; 3 Centre for Medical Image Computing, Department of Medical Physics and Bioengineering, UCL, London, UK; 4 Cancer Research UK Cancer Imaging Centre, The Institute of Cancer Research (ICH) and Royal Marsden NHS Foundation Trust (RHM), London, UK; 5 Centre for Public Health Research, Massey University, Wellington, New Zealand; 6 School of Social and Community Medicine, University of Bristol, Bristol, UK

**Keywords:** ALSPAC, Birthweight, Breast density, In utero, Magnetic resonance imaging, Mammographic density, Maternal, Mediation analysis, Pre-natal, Systematic review

## Abstract

**Background:**

Breast density, the amount of fibroglandular tissue in the adult breast for a women’s age and body mass index, is a strong biomarker of susceptibility to breast cancer, which may, like breast cancer risk itself, be influenced by events early in life. In the present study, we investigated the association between pre-natal exposures and breast tissue composition.

**Methods:**

A sample of 500 young, nulliparous women (aged approximately 21 years) from a U.K. pre-birth cohort underwent a magnetic resonance imaging examination of their breasts to estimate percent water, a measure of the relative amount of fibroglandular tissue equivalent to mammographic percent density. Information on pre-natal exposures was collected throughout the mothers’ pregnancy and shortly after delivery. Regression models were used to investigate associations between percent water and pre-natal exposures. Mediation analysis, and a systematic review and meta-analysis of the published literature, were also conducted.

**Results:**

Adjusted percent water in young women was positively associated with maternal height (*p* for linear trend [*p*_t_] = 0.005), maternal mammographic density in middle age (*p*_t_ = 0.018) and the participant’s birth size (*p*_t_ < 0.001 for birthweight). A 1-SD increment in weight (473 g), length (2.3 cm), head circumference (1.2 cm) and Ponderal Index (4.1 g/cm^3^) at birth were associated with 3 % (95 % CI 2–5 %), 2 % (95 % CI 0–3 %), 3 % (95 % CI 1–4 %) and 1 % (95 % CI 0–3 %), respectively, increases in mean adjusted percent water. The effect of maternal height on the participants’ percent water was partly mediated through birth size, but there was little evidence that the effect of birthweight was primarily mediated via adult body size. The meta-analysis supported the study findings, with breast density being positively associated with birth size.

**Conclusions:**

These findings provide strong evidence of pre-natal influences on breast tissue composition. The positive association between birth size and relative amount of fibroglandular tissue indicates that breast density and breast cancer risk may share a common pre-natal origin.

**Electronic supplementary material:**

The online version of this article (doi:10.1186/s13058-016-0751-z) contains supplementary material, which is available to authorized users.

## Background

Recent meta-analyses and pooled analyses [1, 2] have identified positive associations between birth size and breast cancer risk, suggesting that the pre-natal period may be a critical time window of exposure for risk of breast cancer later in life. The mechanisms linking birth size to risk are not known, but birth size may be a correlate of in utero exposures to mitogens [1]. Such mitogens may influence the size of the stem cell pool in the embryonic breast, which, in turn, affects the development of the gland at puberty and, ultimately, breast cancer risk later in life [3]. If true, such a hypothesis would suggest that the association between pre-natal exposures and breast cancer risk may be mediated, at least in part, by influences on breast tissue composition in early adulthood.

Mammographic percent density, which reflects variations in the relative amounts of fat and fibroglandular tissue in the breast given a woman’s age and body mass index (BMI), is a strong breast cancer risk factor [4]. Mammographic percent density is highest at young ages, when susceptibility to breast carcinogens is greatest, and tracks through a woman’s adult life [5]. Despite evidence that mammographic percent density may be established early in life, few studies have assessed the association of birth size and other pre-natal exposures with mammographic percent density. Existing studies have been restricted to middle-aged and older women, as the risk of radiation-induced breast cancer precludes the use of mammography at younger ages [6]. Consequently, there has been no investigation of the role of pre-natal exposures in young women (i.e., prior to the breast tissue being affected by reproductive-related events).

In the present study, we investigated the relationship between prospectively collected data on a wide range of pre-natal exposures, including birth size, and breast tissue composition, as assessed by ionising radiation-free magnetic resonance imaging (MRI), in nulliparous young women within a British pre-birth cohort, and we conducted a systematic review of the relevant published literature.

## Methods

### Study population

The Avon Longitudinal Study of Parents and Children (ALSPAC) is a prospective pre-birth cohort of 14,775 children born in Avon, England (representing 72 % of the eligible population [7]), between 1 April 1991 and 31 December 1992 [7, 8]. For this study, young, nulliparous women born from singleton pregnancies who regularly participated in follow-up surveys and had never been diagnosed with cancer or a hormone-related disease were invited to attend an MRI examination of their breasts at the University of Bristol Clinical Research and Imaging Centre between June 2011 and November 2014. Women who had contraindications for MRI (e.g., pregnancy, metal implants) were excluded. Of the 2530 potentially eligible women invited, 500 (19.8 %) attended. The low response rate reflected the highly demanding nature of the study, as well as relocation away from the study area (e.g., to attend university). However, participants were similar to potentially eligible women who did not participate in relation to socio-demographic factors and body size measurements (e.g., mean birthweight and BMI at age 16 years were 3390.9 g [SD 21.6 g] and 21.2 kg/m^2^ [SD 0.2 kg/m^2^], respectively, amongst women who participated, and 3397.4 g [SD 11.4 g] and 21.5 kg/m^2^ [SD 0.1 kg/m^2^], respectively, amongst those who did not). Mothers of participants provided access to their mammograms taken as part of the U.K. national screening programme if they were in the targeted age group (50–70 years).

The study received approval from all relevant ethics committees (listed below in the Ethics approval and consent to participate subsection). Participants provided written informed consent.

### Data collection

Information on maternal, in utero and birth size variables was collected from self-administered maternal questionnaires at enrolment in early gestation, throughout pregnancy and shortly after delivery, supplemented by obstetric and paediatric records [9]. During their MRI examinations, participants completed a short questionnaire on menstruation-related variables, and anthropometric measurements were taken. The mother’s BMI, parity and menopausal status closest to the time of mammography were obtained through face-to-face clinical assessments and self-completed questionnaires. The study website contains details of all the data that are available through a fully searchable data dictionary [9].

### Breast tissue composition assessment

Young women underwent an examination using a 3-T Siemens MAGNETOM Skyra MRI system (Siemens Healthcare, Erlangen, Germany) with a breast coil that surrounded both breasts and with the women in prone position. For each woman, three sets of images through both breasts were obtained: (1) T1-weighted VIBE 3-D images (approximately 176 images per woman) with a voxel size of 0.76 × 0.76 × 0.90 mm^3^, (2) T2-weighted transaxial images (approximately 40 images per woman) with in-plane resolution of 0.85 × 0.85 mm^2^ and slice thickness of 4 mm, and (3) sagittal Dixon images (between 37 and 44 per woman) with in-plane resolution 0.74 × 0.74 mm^2^ and slice thickness of 7.7 mm. Fully automated algorithms were developed to estimate breast volume using both T1-weighted and T2-weighted images and perform fat/water segmentation on T2-weighted images, whilst semi-automated breast and fat/water segmentation methods were developed for the Dixon images (details provided in Additional file 1: Methods 1). These algorithms yielded left-right average estimates of volumes (in cubic centimetres) of breast, water and fat (the latter two correspond to mammographic dense and non-dense tissues, respectively), as well as percent water. Percent water has been shown to be highly correlated with mammographic percent density of the same women [10–12]. In comparisons in a random sample of 200 participants, we found little difference in breast measurements across the different MRI images (Additional file 1: Methods 1), and results from T1-weighted and T2-weighted images are presented here. Valid breast parameters were obtained for 491 of the 500 participants who underwent the MRI examination.

Processed digital mammographic images were successfully retrieved from screening centres for 175 mothers. Left and right craniocaudal images were read using the Cumulus semi-automated area-based method [13, 14] to estimate average breast, non-dense and dense areas (in square centimetres), and percent density (mammographic percent density). Cumulus density readings of processed images are strong predictors of breast cancer risk [15]. Readings were performed by a single observer (IdSS) who was blind to the women’s characteristics (within-observer intra-class correlation 0.92).

### Statistical analysis

Linear regression models were fitted to examine associations between participants’ breast tissue parameters and maternal, in utero and birth size variables. Breast tissue parameters were first log-transformed to achieve near-normal distributions. To improve interpretability, exponentiated estimated regression parameters are reported; these represent the relative percent change (RC) in breast measurements associated with a unit increase in the exposure of interest. Continuous exposure measurements were standardised and, where appropriate, grouped into relevant categories or using quartiles as cut-off points. Exposure effects were adjusted for (1) age, BMI, phase of menstrual cycle (luteal, follicular and irregular period), hormonal contraceptive use at the time of MRI (as described in Table 1), and, when investigating the role of maternal mammographic density measurements, also for maternal age and BMI at mammography; and (2) further adjusted for other maternal, in utero or birth size variables as specified in the tables and figures. For simplicity, variables (1) and (2) will be referred to hereafter as minimally and mutually adjusted effects, respectively.

**Table 1:** Selected characteristics of the participants and their mothers

	*n*	Mean %	SD	Median	IQR
Participant characteristics at MRI examination					
Age, months	491	257.9	11.0	259.0	14.0
BMI, kg/m^2^	487	23.9	4.4	23.0	5.1
Menstrual cycle^a^					
Luteal phase	70	14.4			
Irregular periods	50	10.3			
Follicular phase	28	5.8			
Use of hormonal contraception	339	69.6			
Left-right average breast volume, cm^3^	490	647.2	461.1	507.8	469.2
Left-right average breast fat volume, cm^3^	490	406.3	349.5	292.2	327.9
Left-right average breast water volume, cm^3^	490	240.9	131.2	209.8	172.4
Left-right average breast percent water,^b^ %	491	41.8	10.3	41.7	16.0
Maternal characteristics at participant’s birth					
Mother’s age at menarche, years	449	12.9	1.5	13.0	2.0
Mother ever used oral contraceptive pill, %	452	96.7			
Age when mother first used contraceptive pill, years	435	18.8	3.1	18.0	3.0
Mother’s height, cm	446	16.6	6.5	165.1	7.6
Mother’s age at first birth, years	463	26.5	4.7	27.0	7.0
Mother’s age at participant’s birth, years	467	29.9	4.5	30.0	6.0
Mother’s parity at participant’s birth					
0	223	48.5			
1	161	35.0			
2+	76	16.5			
Mother’s pre-pregnancy BMI, kg/m^2^	430	22.3	3.0	21.7	3.5
Maternal history of BC when participant was 8 years old, %	355	11.6			
Maternal characteristics at mammography					
Age, years	176	52.7	3.9	52.0	5.0
BMI,^c^ kg/m^2^	165	24.3	4.7	23.3	5.4
Left-right average breast area, cm^2^	176	295.0	141.3	266.8	167.1
Left-right average dense area, cm^2^	176	63.9	37.3	59.3	37.6
Left-right average percent density, %	176	25.3	13.4	24.8	20.4
In utero exposures					
Placental weight, g	121	587.1	132.9	580.0	160.0
Absolute GWG, week 0 to delivery, kg	422	12.1	3.9	12.0	5.0
Mother drank alcohol during pregnancy, %	459	74.1			
Mother smoked during pregnancy, %	464	10.6			
Participant characteristics at birth					
Birthweight, g	460	3395.0	472.6	3400.0	565.0
Birth length, cm	362	50.5	2.3	50.8	2.7
Head circumference, cm	370	34.6	1.2	34.6	1.5
PI,^d^ g/cm^3^	358	26.4	4.1	26.1	3.2
Gestational age,^e^ weeks					
< 39	93	19.9			
39	101	21.6			
40	132	28.3			
≥ 41	141	30.2			

*Abbreviations: MRI* Magnetic resonance imaging, *BC* Breast cancer, *BMI* Body mass index, *GWG* Gestational weight gain, *PI* Ponderal Index

^a^Estimated for women not using hormonal contraception by calculating the number of days since the last menstrual period (date of MRI to start of last menstrual period). Luteal (days 14–17 to 28–31) and follicular (days 0 to 14–17) phases and an ‘irregular period’ (32+ days) were defined using average length of menstrual cycle

^b^Sections of the breast were missing in the MRI images for one participant; thus, volumetric measurements could not be ascertained, and percent water only was used

^c^Clinically measured or self-reported BMI. Median time interval between BMI assessment and mammography was 3 years (IQR 1.5 years)

^d^PI defined as birthweight (g)/birth length (cm^3^)

^e^Data available only as a categorical variable

Mediation analyses were performed to investigate separately whether the effect of maternal exposures on the participants’ percent water were mediated via birth size, and whether the effect of birth size was mediated through adult height and BMI [16]. Linear regression models were fitted to the percent water, the exposure and each mediator in turn, with relevant confounders included and interactions between each exposure-mediator pair investigated. Results are presented in terms of direct (i.e., not mediated) and indirect (i.e., mediated) effects and expressed as percent changes, with 95 % CIs for the indirect effects obtained by bootstrapping [17].

Sensitivity analyses were conducted using (1) an alternative method to estimate percent water on Dixon images (Additional file 1: Methods 1) and (2) multiple imputation to deal with missing exposure and confounder data under the missing-at-random assumption [18] to obtain results based on all participants with valid MRI breast parameters (*n* = 491). Imputation by chained equations method was used, including all the exposures, confounding factors and outcomes involved in the analysis. The models described above were fitted to each of 20 imputed datasets, and overall estimates were obtained using Rubin’s rules [19].

Data analysis was conducted using STATA version 14 software (StataCorp, College Station, TX, USA). All tests of significance were two-sided.

### Systematic review of pre-natal exposures and breast tissue composition

The original protocol and methodology of the review are given in Additional file 1: Methods 2 and 3. Briefly, a search for studies published between 1 January 1970 and 25 September 2015 was conducted in PubMed using the search terms detailed in Additional file 1: Methods 3. Paper screening and data extraction were completed independently by two reviewers (RD, IdSS). The quality of the eligible papers was assessed by developing a standardised quality score (ranging from 0 [lowest quality] to 59 [highest quality]) based on 15 individual parameters reflecting the potential for selection bias, measurement error and confounding (Additional file 1: Methods 3).

Estimates of association between pre-natal exposures and breast density, as ascertained by mammography or an alternative approach, were extracted from each study and reported graphically using forest plots, whenever appropriate. To summarise the results in terms of linear trends, we first estimated linear effects across categorical exposures using study-specific weighted regression across the reported regression coefficients (with weights proportional to their SE). Derived study-specific linear trend coefficients and study-specific linear effects of continuous exposures were then summarised using random effects meta-analysis. Between-study heterogeneity was assessed using the *I* ^2^ statistic. To examine potential sources of heterogeneity, study-specific trend coefficient estimates were stratified according to variables defined a priori (i.e., menopausal status at mammography, source of pre-natal exposure data, breast density assessment method). Funnel plots and Egger’s test were used to assess publication bias.

## Results

### Study subjects

Table 1 presents the distributions of maternal, in utero and birth size characteristics of the participants. The mean age of the participants at the time of MRI was 21.5 years. Relative to mothers for whom mammograms were not available, those with mammograms were, as expected given the age group targeted by the U.K. national breast screening programme, older (31.7 vs. 28.8 years) and more likely to have older children (55.6 % vs. 49.3 %) at the participants’ birth. There were no differences, however, in the participants’ characteristics according to whether a mammogram could be retrieved for their mothers (data not shown). Participants’ percent water was inversely associated with BMI at the time of MRI, but not with age (reflecting their rather narrow age range) or menstrual phase/contraceptive use (Additional file 2: Table S1). Mothers’ mammographic percent density was inversely associated with both their age and their BMI at the time of mammography, but not with parity or menopausal status (Additional file 2: Table S1).

### Pre-natal exposures and MRI breast tissue measurements

**Fig. 1:**
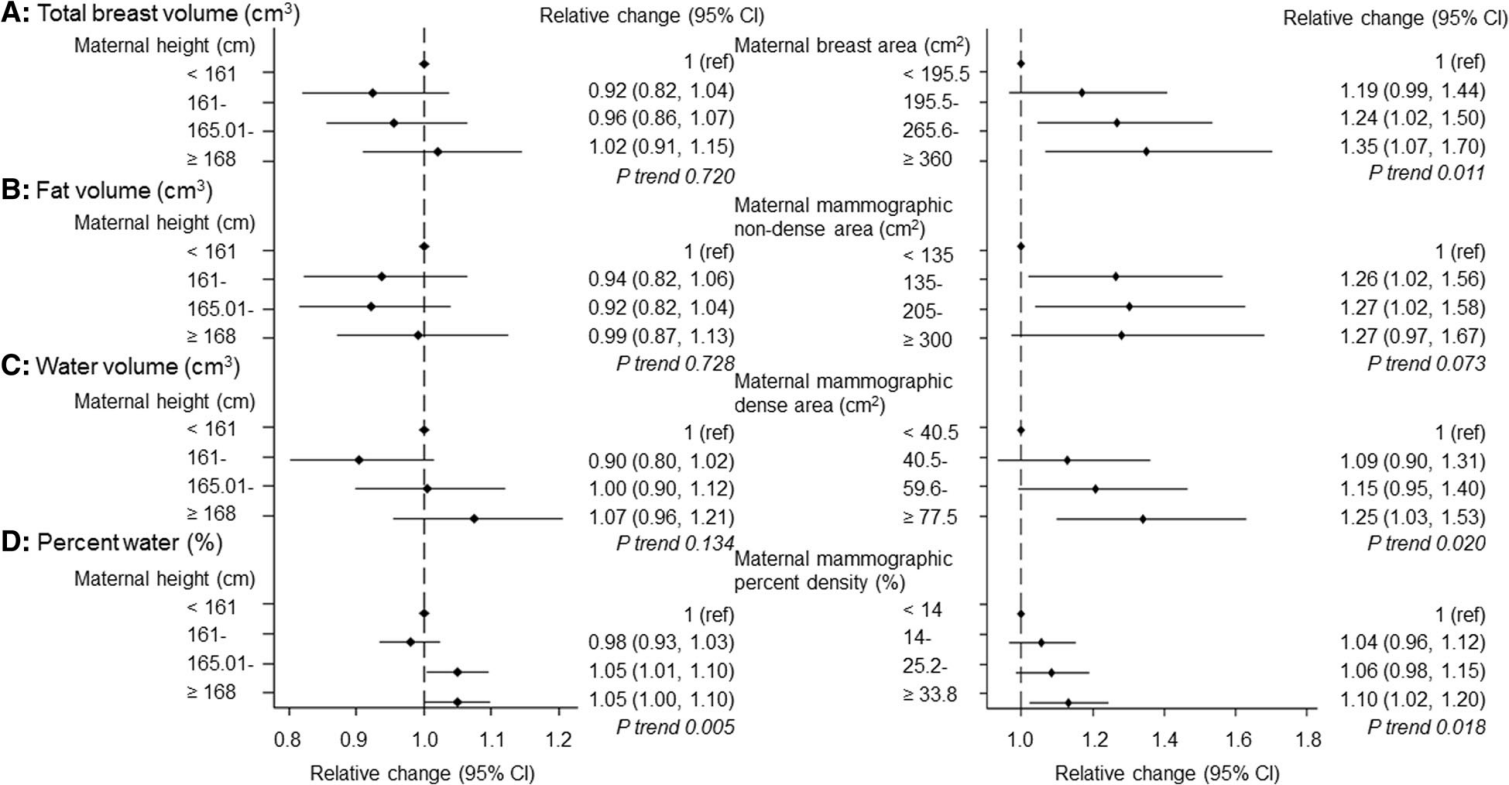
Magnetic resonance imaging (MRI)-based breast tissue measurements in relation to maternal height and maternal mammographic breast measurements (minimally adjusted estimates). *ref* Reference category. MRI breast measurements were log-transformed, and exponentiated estimated regression parameters, with 95 % CI calculated by exponentiating the original 95 % CIs, are presented. Models were adjusted for the participant’s age, BMI and menstrual phase/hormonal contraceptive use at the time of MRI and, where appropriate, mother’s age and BMI at mammography. Continuous variables were centred at the mean

In univariate analyses, percent water in daughters was positively correlated with maternal mammographic percent density (*r* = 0.23; *p* = 0.003; *n* = 175), mirroring positive daughter-mother correlations in the amounts of dense tissue (*r* = 0.18; *p* = 0.014) (Additional file 3: Figure S1). Both maternal height and mammographic percent density were positively associated with the participants’ percent water in minimally (Fig. 1d) and mutually (Table 2) adjusted analyses. One SD increment in maternal height and mammographic percent density were associated, respectively, with a 2 % (RC 1.02; 95 % CI 1.00–1.04) and a 6 % (RC 1.06; 95 % CI 1.01–1.10) increase in percent water (Table 2), reflecting mainly increases in water volume (Fig. 1c). No associations with other maternal characteristics or in utero exposures were observed (Table 2).

**Table 2:** Mutually adjusted associations of MRI percent water in daughters with maternal characteristics and markers of in utero exposures estimated using the complete and imputed data

	Relative change in MRI percent water, geometric mean (95 % CI)	
	Complete data^a^	Imputed data^b^
Maternal characteristics^c^	*n* = 303	*n* = 490
At participant’s birth only		
Mother’s age at menarche (per 1 SD 1.5 years)	0.98 (0.96–1.01)	1.00 (0.98–1.01)
Age mother first used OC (per 1 SD 3.1 years)	1.00 (0.98–1.02)	1.00 (0.98–1.02)
Mother’s height (per 1 SD 6.5 cm)	**1.02 (1.00–1.04)**	**1.03 (1.01–1.05)**
Mother’s age at first birth (per 1 SD 4.7 years)	0.99 (0.96–1.03)	0.98 (0.96–1.01)
Mother’s age at participant’s birth (per 1 SD 4.5 years)	1.00 (0.96–1.03)	1.01 (0.98–1.03)
Mother’s pre-pregnancy BMI (per 1 SD 3.0 kg/m^2^)	1.01 (0.99–1.03)	1.01 (0.99–1.03)
Mother’s parity at participant’s birth		
0	1 (ref)	1 (ref)
1	1.04 (0.99–1.10)	1.02 (0.98–1.06)
2+	1.00 (0.93–1.08)	1.00 (0.95–1.06)
Mother had a history of breast cancer		
No	1 (ref)	1 (ref)
Yes	1.01 (0.95–1.08)	1.01 (0.96–1.07)
Maternal characteristics at participant’s birth and at mammography^d^		
Mother’s MPD (per 1 SD 13.4 %)	**1.06 (1.01–1.10)**	–
In utero exposures^e^	*n* = 107	*n* = 490
Placental weight (per 1 SD 133.5 g)	1.03 (0.99–1.07)	1.03 (0.99–1.07)
Absolute GWG, week 0 to delivery (per 1 SD 3.9 kg)	0.97 (0.93–1.01)	1.00 (0.98–1.02)
Mother drank alcohol during pregnancy (%)		
No	1 (ref)	1 (ref)
Yes	1.01 (0.91–1.11)	1.00 (0.95–1.06)
Mother smoked during pregnancy (%)		
No	1 (ref)	1 (ref)
Yes	0.95 (0.89–1.03)	0.99 (0.96–1.04)

*Abbreviations: MRI* Magnetic resonance imaging, *BMI* Body mass index, *GWG* Gestational weight gain, *OC* Oral contraceptives, *MPD* Mammographic percent density *ref* Reference category

MRI breast measurements were log-transformed, and exponentiated estimated regression parameters, with 95 % CIs calculated by exponentiating the original 95 % CIs, are presented. ﻿Bold indicates 95 % CI do not cross the null (1.00)

^a^Analysis restricted to those with non-missing data for all variables included in the models

^b^See Statistical methods section in main text

^c^Maternal and confounding factors (age, BMI and menstrual phase/hormonal contraceptive use at MRI) were included in the model simultaneously

^d^Analysis restricted to the subset of participants for whose mothers it was possible to retrieve a mammogram (*n* = 116). Model includes all the maternal characteristics at the participant’s birth listed in the table as well as maternal MPD in later life (mean age at mammography 52.8 years; Table 1), adjusting for the daughters’ age, BMI and menstrual phase at the time of MRI and for the mothers’ age and BMI at the time of mammography

^e^In utero and confounding factors (age, BMI and menstrual phase/hormonal contraceptive use at the time of MRI) were included in the model simultaneously

**Fig. 2:**
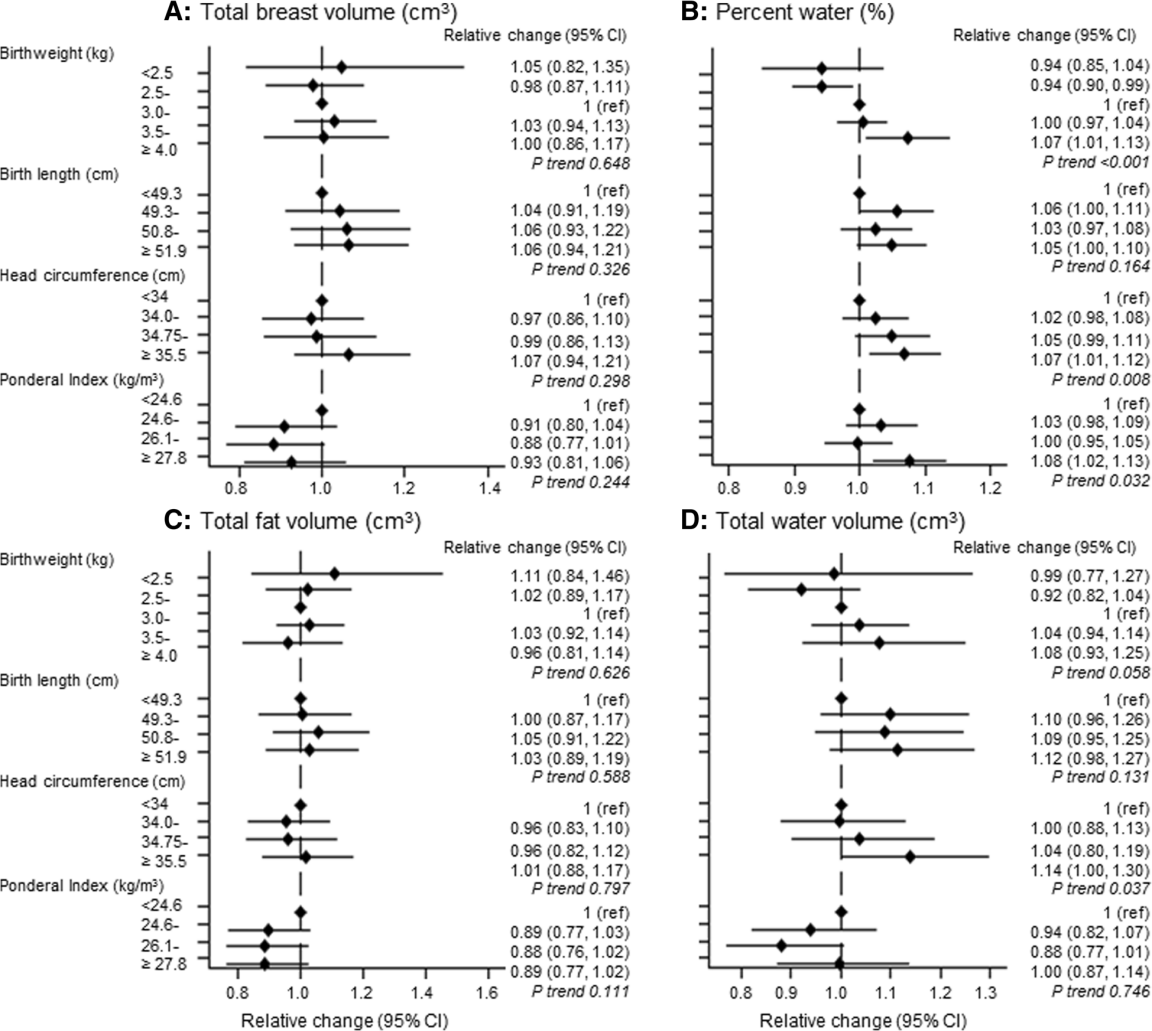
Magnetic resonance imaging (MRI) breast tissue measurements in relation to the participant’s size at birth (minimally adjusted estimates). *ref* Reference category. MRI breast measurements were log-transformed, and exponentiated estimated regression parameters, with 95 % CIs calculated by exponentiating the original 95 % CIs, are presented. Models are adjusted for the participant’s age, body mass index (BMI) and menstrual phase/hormonal contraceptive use at the time of MRI and, where appropriate, mother’s age and BMI at the time of mammography. Continuous variables were centred at the mean

Weight, length, head circumference and ponderal index at birth were all associated with percent water in minimally adjusted analysis (Fig. 2b), reflecting similar positive associations with water volume, with the exception on ponderal index, (Fig. 2d). A 1-SD increment in weight (473 g), length (2.3 cm), head circumference (1.2 cm) and ponderal index (4.1 g/cm^3^) at birth was associated with a minimally adjusted RCs of 1.03 (95 % CI 1.02–1.05), 1.02 (1.00–1.03), 1.02 (1.01–1.04) and 1.01 (1.00–1.03), respectively. For birthweight, this corresponded to an absolute 5.45 % (95 % CI 1.05–9.85) difference in minimally adjusted mean percent water between the extreme categories of the birthweight distribution (i.e., ≥4.0 vs. <2.5 kg) (Additional file 3: Figure S2). Gestational age was not associated with percent water (Additional file 2: Table S2); indeed, further adjustment for this variable did not materially affect the magnitude of the birth size-percent water association (Table 3).

**Table 3:** Associations between participant’s size at birth and MRI percent water

			Relative change in MRI percent water, geometric means^a^ (95 % CI)	
			Complete data^b^	Imputed data^c^ (*n* = 491)
Absolute size vs. rate of growth			*n* = 455	
Model 1	Birthweight (per 1 SD 472.6 g)		**1.03 (1.02–1.05)**	**1.03 (1.02–1.05)**
Model 2	Birthweight (per 1 SD 472.6 g)		**1.04 (1.02–1.06)**	**1.04 (1.02–1.06)**
	Gestational age (weeks)	<39	1 (ref)	1 (ref)
		39	0.97 (0.92–1.02)	0.97 (0.92–1.02)
		40	0.97 (0.92–1.02)	0.97 (0.92–1.02)
		41+	0.96 (0.92–1.01)	0.96 (0.92–1.01)
LR test/Wald test *p* value^d^			0.519	0.477
Which measure best captures linear (skeletal) growth?			*n* = 356	
Birth length (per 1 SD 2.3 cm)			1.00 (0.98–1.02)	1.01 (0.99–1.03)
Head circumference (per 1 SD 1.2 cm)			**1.02 (1.00–1.05)**	**1.02 (1.00–1.04)**
Linear growth vs. adiposity			*n* = 361	
Model 1	Birthweight (per 1 SD 472.6 g)		**1.03 (1.01–1.05)**	**1.03 (1.02-1.05)**
Model 2	Birthweight (per 1 SD 472.6 g)		**1.03 (1.00–1.06)**	**1.03 (1.01–1.06)**
	Head circumference (per 1 SD 1.2 cm)		1.01 (0.98–1.03)	1.00 (0.97–1.03)
LR test/Wald test *p* value^d^ (*n* = 353)			0.671	0.917
Model 1	Birthweight (per 1 SD 472.6 g)		**1.03 (1.01–1.05)**	**1.03 (1.02–1.05)**
Model 2	Birthweight (per 1 SD 472.6 g)		**1.03 (1.01–1.05)**	**1.03 (1.02–1.05)**
	Ponderal Index (per 1 SD 4.1 g/cm^3^)		1.01 (0.99–1.02)	1.00 (0.99–1.02)
LR test/Wald test *p* value^d^			0.577	0.654

*Abbreviations: MRI* Magnetic resonance imaging, *LR* Likelihood ratio test, *ref* Reference category

^a^MRI percent water was log-transformed for the analysis, and exponentiated estimated regression parameters, with 95 % CIs calculated by exponentiating the original 95 % CIs, are presented. Models adjusted for age, BMI *z*-score and menstrual phase/hormonal contraceptive use at the time of MRI scan. Bold indicates 95 % CI do not cross the null (1.00)

^b^Analysis restricted to those with non-missing data for all variables included in each model

^c^See Statistical methods section of main text

^d^LR test performed on the complete record data, while a Wald test was performed on the imputed data (and summarised using Rubin’s rule), to test the null hypothesis that the inclusion of the additional variable in model 2 did not improve the fit to the data

Weight and length at birth were correlated with each other (*r* = 0.67, *p* < 0.0001), and both were correlated with head circumference (*r* = 0.71, *p* < 0.0001, and *r* = 0.50, *p* < 0.0001, respectively) and ponderal index (*r* = 0.29, *p* = 0.001, and *r* = 0.46, *p* = 0.001, respectively). Both length and head circumference reflect linear (skeletal) growth, but in mutually adjusted analysis only the association of the latter with percent water persisted (Table 3). Ponderal index reflects adiposity, while birthweight is a function of both linear growth and adiposity; however, only the birthweight-percent water association persisted when head circumference or ponderal index was included in the model (Table 3).

### Sensitivity analyses

Similar findings were observed in multiple imputation analyses or when the breast tissue measurements were estimated using an alternative method to measure percent water on Dixon images (Tables 2 and 3 and Additional file 2: Table S2).

### Mediation analyses

**Fig. 3:**
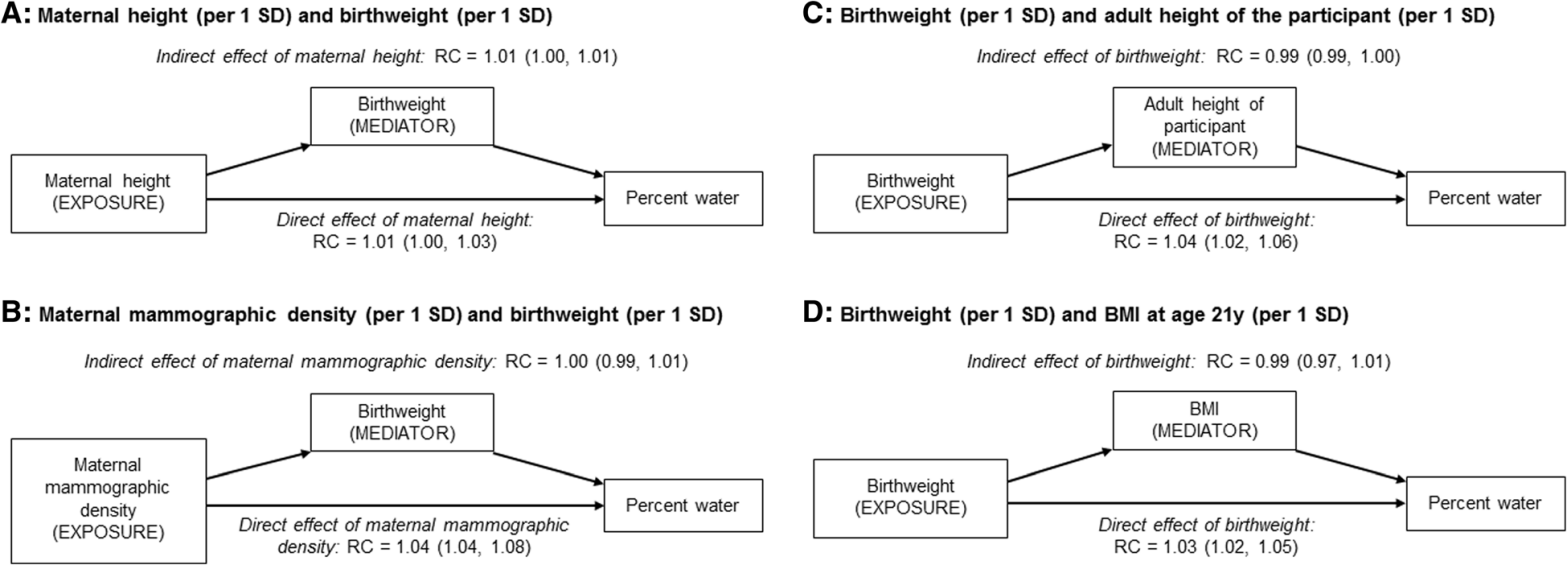
Indirect and direct effects (relative change in geometric mean) of **a**) maternal height and **b**) maternal mammographic density accounting for the mediating effect of birthweight, and birth weight accounting for the mediating effect of **c**) height and **d**) BMI at age 21 years, on MRI percent water. *RC* Relative percent change, *BMI* Body mass index. MRI-based percent water was log-transformed for the analysis, and exponentiated estimated regression parameters, with 95 % CIs calculated by exponentiating the original 95 % CIs, are presented. Model shown in (**a**) was adjusted for age, BMI and menstrual phase//hormonal contraceptive use at the time MRI, maternal education level, pre-pregnancy BMI, and smoking during pregnancy. Model shown in (**b**) was adjusted as for (**a**) plus maternal height, and maternal age and BMI at the time of mammography. Model shown in (**c**) was adjusted for age, BMI and menstrual phase/hormonal contraceptive use at the time of MRI, maternal education level, height, and smoking during pregnancy. Model shown in (**d**) was adjusted for age, BMI (linear and quadratic term), and menstrual phase/hormonal contraceptive use at the time of MRI

The association between maternal height and the participants’ percent water was partly mediated by birthweight, with about half of its total effect (mutually adjusted total effect RC = 1.01; 95 % CI 1.00–1.01) being attributable to its influence on birthweight and the effect of birthweight on percent water (indirect effect 1.01; 95 % CI 1.00–1.01) (Fig. 3a). For the birthweight-percent water association (mutually adjusted total effect 1.03; 95 % CI 1.02–1.05), there appears to be some evidence of protective mediation through the adult height of the participants (indirect effect RC = 0.99; 95 % CI 0.99–1.00). In contrast, there was no evidence of mediation of the effect of maternal mammographic percent density on the participants’ percent water through birthweight, nor was there evidence of the effect of birthweight on percent water via BMI at the time of MRI (Fig. 3b and d).

### Systematic review

In the systematic search, we identified 208 abstracts, of which 12 were eligible (Additional file 3: Figure S3). Of these, most were conducted with post-menopausal women, although four included or were restricted to pre-menopausal participants [20–23]. Two studies included women in young adulthood [12, 24], but these contributed data only to the mothers-daughters breast density analysis (Additional file 2: Table S3 and S4). These two studies [12, 24] were also the only ones not to use mammography to assess participants’ breast density. All but two studies adjusted for age, BMI and, if appropriate, also menopausal status at breast density assessment [21, 25].

**Fig. 4:**
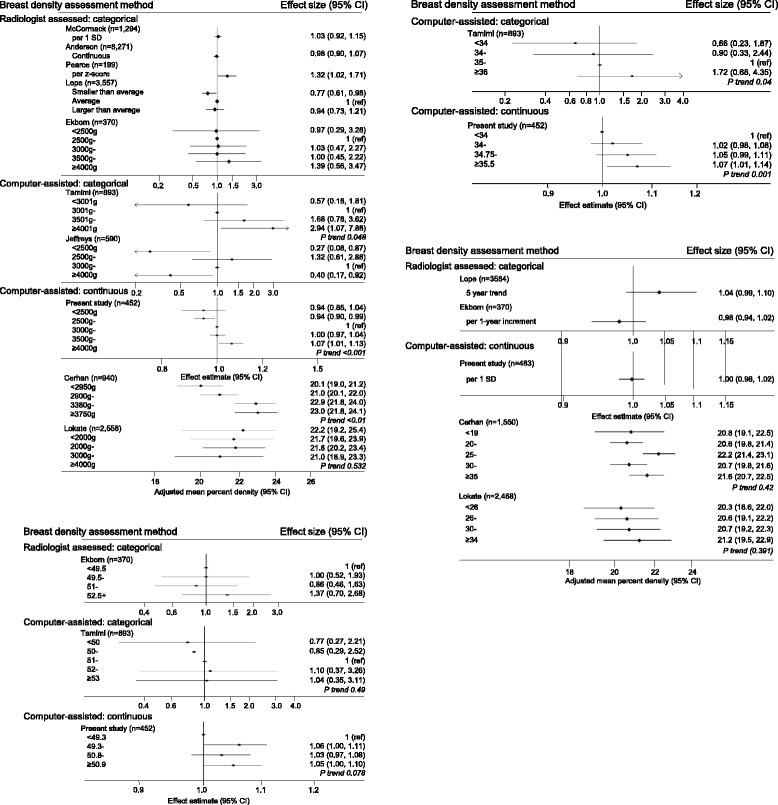
Systematic review of studies investigating (**a**) birth size measurements and (**b**) maternal age and percent breast density. I. Birthweight and percent density, II. birth length and percent density, and III. head circumference and percent density data are shown. Studies classified as using a computer-assisted categorical breast density assessment method collected a quantitative measure of mammographic percent density but used a categorical measure in the analysis

Nine studies investigated associations between birth size measurements and adult breast density. The meta-analysis of their study-specific trend estimates, including those derived from the present study, revealed positive trends in the relative amount of fibroglandular tissue in the breast, as assessed by mammographic percent density or percent water, with birth size, albeit with high between-study heterogeneity (Fig. 4a and Table 4). Analysis by potential source of heterogeneity showed that this positive trend was stronger among studies assigned a high overall quality score, and in particular among those that relied on less error-prone birth size data from hospital records and more objective computer-assisted density measurements (Table 4). The positive trend in percent breast density with birth size was found in analyses restricted to study-specific estimates derived from pre- or post-menopausal women only, albeit more marked for the latter (Table 4). Similar results were observed when estimates derived from the present study were excluded. There was evidence of publication bias (*p*<0.001), which disappeared (*p* = 0.928) when the Tamimi study [26] was excluded (Additional file 3: Figure S4).

**Table 4:** Meta-analysis of the association between various birth size measurements and percent breast density, stratified by potential sources of between-study heterogeneity

Perinatal factor	Number of studies^a^	Average relative change^b^ (95 % CI)	*z*-score *p* value	*I* ^2^ statistic (%)	References
Birth size measurements:					
Birthweight					
Overall^c^	9	1.59 (1.58–1.59)	<0.001	100.0	[21, 22, 25, 26, 33, 35, 49, 50]; present study
Using Andersen et al. [35] OR2^d^	9	1.59 (1.58–1.59)	<0.001	100.0	
Excluding present study	8	1.63 (1.62–1.63)	<0.001	100.0	
Menopausal status					
Pre-menopausal women	3	1.03 (1.02–1.04)	<0.001	38.1	[21, 22]; present study
Post-menopausal women	3	1.72 (1.71–1.72)	<0.001	99.7	[21, 22, 26]
Source of birthweight data					
Self-/parent report	4	0.99 (0.99–1.00)	0.117	91.0	[21, 22, 35, 50]
Hospital records	5	1.67 (1.66–1.67)	<0.001	10.0	[25, 26, 33, 49] present study
Breast density assessment method					
Radiographer-assessed	4	1.05 (1.00–1.10)	0.066	49.7	[25, 33, 35, 49]
Computer-assisted	5	1.59 (1.58–1.59)	<0.001	100.0	[21, 22, 26, 50] present study
Restricted to hospital records					
Radiographer-assessed	3	1.07 (1.02–1.13)	0.013	33.3	[25, 33, 49]
Computer-assisted	2	1.67 (1.66–1.67)	<0.001	100.0	[26]; present study
Quality score^e^					
Highest tertile (≥50)	3	1.67 (1.66–1.67)	<0.001	100.0	[26, 35]; present study
Middle tertile (40–50)	3	0.99 (0.99–1.00)	0.142	94.7	[22, 48, 50]
Lowest tertile (<40)	3	1.04 (0.99–1.09)	0.160	26.3	[21, 25, 35]
Birth length					
Overall	3	1.02 (1.00–1.04)	0.051	89.2	[25, 26]; present study
Head circumference					
Overall	2	1.11 (1.11–1.11)	<0.001	100.0	[26]; present study
Maternal age					
Overall	5	1.01 (1.01–1.02)	<0.001	31.7	[20, 22, 25, 50]; present study
Excluding present study	4	1.01 (1.01–1.02)	<0.001	21.9	
Menopausal status					
Pre-menopausal women	3	1.00 (0.99–1.02)	0.539	0.0	[20, 22]; present study
Post-menopausal women	3	1.01 (1.01–1.02)	<0.001	0.0	[20, 22, 50]
Quality score (maximum 59)^d^					
Highest (≥40)	3	1.01 (1.01–1.02)	<0.001	17.4	[22, 50]; present study
Lowest (<40)	2	1.00 (0.97–1.04)	0.898	67.2	[20, 25]

^a^Lope et al. [20] was not included in the birthweight meta-analysis, owing to concerns about the validity of a summary trend measure across the limited number of categories (three groups)

^b^Due to the high between-study heterogeneity in most strata, these average estimates should be interpreted simply as indicators of the direction of the trend in breast density with increasing birth size

^c^Meta-analysis uses OR1 from Andersen et al. [35] as reported in Table S3, which is adjusted for age at screening and birth cohort: OR 0.98; 95 % CI 0.90–1.07

^d^Meta-analysis uses OR2 from Andersen et al. [35] as reported in Table S3, which is adjusted for age at screening, birth cohort and BMI at age 13 years: OR 1.11; 95 % CI 1.02–1.22

^e^Range 0–59; see Methods section of main text and Additional file 1: Methods 3 for description of how study quality scores were developed

Seven studies reported on associations between other pre-natal exposures with adult breast density (Additional file 2: Table S4). Meta-analyses of study-specific estimates derived from five studies, including the present one, revealed a positive trend between maternal age and relative amount of fibroglandular tissue in the breast, which was stronger in analyses restricted to post-menopausal women, with relatively low between study-heterogeneity (Fig. 4b and Table 4). There was some indication of publication bias (*p* = 0.032) (Additional file 3: Figure S4). Meta-analyses were not possible for other pre-natal exposures, owing to the small number of studies and differences in the way these variables were measured or analysed (Additional file 2: Table S4), but qualitative assessment of the evidence did not reveal any consistent associations of percent breast density with maternal parity (based on *n* = 4 studies, including the present one), maternal smoking (*n* = 3) or alcohol intake (*n* = 2) during pregnancy, gestational age (*n* = 5), or placental weight (*n* = 2).

## Discussion

In this unique pre-pregnancy cohort with a wide range of maternal, in utero and birth size measurements, we found evidence of a positive association between birth size and percent breast density, as measured by percent water, in young adult women, which was not mediated by current body size. Birth size has been shown to be positively associated with breast cancer risk in later life in pooled analysis and meta-analysis [1], albeit not in a recent cohort based on self-reported birthweight [27]. Overall, our findings are consistent with the birth size-breast cancer association being explained by foetal growth influences on breast tissue composition. Maternal height and maternal mammographic percent density were also positively correlated with the participants’ percent water, albeit with evidence that the maternal height association was partly mediated through the effect of birth size on percent water.

The magnitude of the birth size association with percent breast density is small but not negligible. A 1 % increase in percent density corresponds to a 2 % increase in breast cancer risk [28]. Assuming that the effect of birthweight is entirely mediated through changes in breast tissue composition, the observed 3 % increase in percent breast density associated with a 1-SD increment in birthweight would translate to a 6 % increase in breast cancer risk, consistent with the 6 % (95 % CI 2–9 %) increase in breast cancer risk associated with a 0.5-kg increase in birthweight reported in a pooled analysis of original individual-level data derived from 32 studies [29]. Such an effect on risk would be similar to effects reported for other established breast cancer risk factors (e.g., 5-cm increase in adult height [30, 31] or 10-g daily alcohol consumption [31]).

### Strengths and limitations of the present study

Strengths of the present study include the unique pre-birth cohort design with a wide range of prospectively collected pre-natal exposure data. Breast tissue measurements were collected from ionising radiation-free MRI examinations, making this the first study to examine the effect of pre-natal influences on breast tissue composition in young adulthood, prior to changes induced by pregnancies and breastfeeding. Objective (fully automated and, hence, observer-independent) volumetric breast tissue composition measurements were taken using a previously developed and evaluated approach [32].

The response rate was low (approximately 20 %), although comparable to a similar MRI breast study [12], but there was no evidence that the participants constituted a biased sample. Data were missing for some variables, but analyses of complete records and imputed datasets produced similar findings.

### Consistency with other studies

Meta-analysis of study-specific estimates derived from all eligible studies identified in the systematic review, together with those derived from the present study, revealed a significant positive trend between birth size and percent breast density, as assessed by mammographic percent density or percent water, albeit with marked between-study heterogeneity. Positive associations between birthweight and percent breast density were reported in studies based on computer-assisted methods to assess breast density [22, 26], but not in those that relied on radiologist-assessed categorical measurements (e.g., Wolfe’s [33] or Boyd’s [34] categories, American College of Radiology Breast Imaging Reporting and Data System (BI-RADS) [35]). Inconsistencies are likely due to the relatively small effect of birth size, which cannot be captured by categorical density classifications (Additional file 3: Figure S2). Analyses stratified by source-of-exposure data showed a significant positive trend between hospital-recorded birthweight measurements and percent breast density, but not in studies that used parental or adult self-reports, consistent with findings derived from a pooled analysis of birth size and breast cancer studies [1].

### Plausibility of the findings

Birth size is a strong predictor of later physical development, with both weight and length at birth being associated with childhood growth, age at menarche [36] and adult body size [37]. Thus, the observed association between birth size and percent breast density may be mediated by childhood and adolescent growth trajectories [26]. Mutually adjusted analysis revealed that birthweight had the strongest independent association with percent water, suggesting that both linear growth and adiposity may affect breast tissue composition later in life. Furthermore, the association of birthweight with percent water did not appear to be mediated primarily via BMI at the time of MRI examination, suggesting that the effect is mainly independent of childhood and adolescent growth. There was some indication of a protective mediation effect of adult height in the association of birthweight with percent water, which may potentially reflect interactions between birth size, post-natal catch-up growth and pubertal development on percent water. Overall, dependent on the strong assumption of no unmeasured confounding, the mediation analysis provides strong evidence of a causal association between birthweight and breast tissue composition.

The observed associations with birth size and percent water mostly reflect associations with water volume, indicating that the amount of fibroglandular tissue in the breast may be set in utero. These findings parallel the effect of birth size on breast cancer risk and provide further support for the hypothesis that the intrauterine environment may play a role in determining both breast tissue composition and breast cancer risk [38, 39]. The pool of breast-specific stem cells, whose size is determined in utero [40], may be a critical factor linking pre-natal exposures to breast tissue composition and breast cancer risk in later life. In utero levels of growth factors (e.g., insulin-like growth factors) and hormones (e.g., sex hormones) are thought to act as mitogens, influencing both the pool size of breast-specific stem cells and possibly birthweight [41], with the former likely to be strongly correlated with the amount of fibroglandular tissue present in the fully developed breast [42].

Maternal height and mammographic density were also positively related to percent water, supporting previous evidence that percent breast density is a highly heritable trait. Twin studies have estimated that an additive genetic model explains 53–60 % of the variance in percent breast density [43, 44]. In the present study, the observed correlation between the participants’ percent water and their mothers’ mammographic percent density was *r* = 0.23 (Additional file 3: Figure S1), consistent with previous studies of mothers and daughters (*r* = 0.25) [12] and dizygotic twin pairs (*r* = 0.27) [45]. Maternal adult height is strongly correlated with daughters’ adult height (in this study, *r* = 0.49), and greater adult height is associated with a higher amount of fibroglandular tissue in the breast [46, 47]. Furthermore, adult height is positively associated with breast cancer risk, and genetic variants and biological pathways affecting adult height play an important role in the aetiology of breast cancer [30]. Maternal height modifies the effect of pregnancy hormones on birthweight [48], and thus it may also affect the risk of breast cancer in daughters through this mechanism.

## Conclusions

Our study, together with the systematic review, provides the strongest evidence so far that pre-natal factors influence breast tissue composition in young adulthood, with the birth size associations with percent breast density paralleling previously reported positive birth size associations with breast cancer risk. Breast density is known to track from middle adulthood [5], but our findings indicate that high-risk women may be identified at an earlier age—a key aspect to consider for prevention [48, 49].

## Additional files

Additional file 1:
**Methods 1.** Validation of breast volume and fat-water segmentation methods using magnetic resonance imaging (MRI) images. **Methods 2.** Protocol of the systematic review on pre-natal exposures and breast tissue composition. **Methods 3.** Systematic literature review of maternal, in utero and birth size variables as well as breast tissue composition. (DOCX 1513 kb)
Additional file 2: Table S1. Mutually adjusted associations of MRI breast tissue measurements in daughters, and mammographic breast measurements in mothers, with age, anthropometry and hormone status at the time of the breast examination. **Table S2.** Minimally adjusted associations of MRI breast percent water in relation to maternal, in utero and birth size characteristics using complete and imputed data (*n* = 491), and Dixon-based MRI breast percent water. **Table S3.** Systematic review of studies investigating the association between birth size measurements, gestational age and percent breast density. **Table S4.** Systematic review of studies investigating the association between maternal and in utero exposures and percent breast density. (DOCX 103 kb)
Additional file 3: Figure S1. Correlation between participants’ MRI breast tissue measurements and their mothers’ mammographic density measurements (*n* = 164). **Figure S2.** Predicted MRI breast percent water geometric means in relation to categories of maternal height, maternal mammographic percent density and participant’s size at birth (minimally adjusted estimates). **Figure S3.** Preferred Reporting Items for Systematic Reviews and Meta-Analyses (PRISMA) flow diagram of the systematic review. **Figure S4. a** and **b** Funnel plots for the meta-analysis of birthweight. **a** Number studies = 9. **b** Number studies = 8. (**c**) Funnel plot for the meta-analysis of maternal age (*n* = 5 studies) and percent breast density. (DOCX 497 kb)

## Abbreviations

ALSPAC: Avon Longitudinal Study of Parents and Children
BC: Breast cancer
BI-RADS: American College of Radiology Breast Imaging Reporting and Data System
BMI: Body mass index
GWG: Gestational weight gain
LR: Likelihood ratio
MPD: Mammographic percent density
MRI: Magnetic resonance imaging
OC: Oral contraceptive
PRISMA: Preferred Reporting Items for Systematic Reviews and Meta-Analyses
RC: Relative percent change
