# Supplementary material for: Pre-natal exposures and breast tissue composition: findings from a British pre-birth cohort of young women and a systematic review
Source: Breast Cancer Res. 2016 Oct 12;18:102. doi: 10.1186/s13058-016-0751-z (PMC5059986; doi:10.1186/s13058-016-0751-z)
Supplement: Additional file 1: — Methods 1. Validation of breast volume and fat-water segmentation methods using magnetic resonance imaging (MRI) images. Methods 2. Protocol of the systematic review on pre-natal exposures and breast tissue composition. Methods 3. Systematic literature review of maternal, in utero and birth size variables as well as breast tissue composition. (DOCX 1513 kb) [file 13058_2016_751_MOESM1_ESM.docx]

**Supporting Methods 1. Validation of breast volume, and fat-water segmentation methods using magnetic resonance imaging (MRI) images**

For each participant, three sets of images were acquired in the sagittal plane (Figure 1): A. multi-slice Dixon images with in-plane resolution 0.74 x 0.74 mm^2^ and slice thickness of 7.7 mm (a phantom test object was developed and used to calibrate the water and fat volumes); B. T1-weighted (T1-w) VIBE 3-D images, with a voxel size of 0.76 x 0.76 x 0.90 mm^3^; and C. multi-slice T2-weighted (T2-w) trans-axial images, with in-plane resolution 0.85 x 0.85 mm^2^ and slice thickness of 4 mm.

Breast measurements were generated from the Dixon method, T1-weight VIBE (T1-w), and T2-weighted trans-axial (T2-W) images (Figure 1) (1).

Two different approaches were used for breast volume and fat/water segmentation. Firstly, a semi-automated breast volume segmentation method using a fuzzy C-means algorithm was applied to the Dixon images of each participant. Misclassified regions outside and within the breast were removed by morphological and manual post-processing editing. For the fat-water segmentation, a modified, semi-automated Dixon-based method was applied. Readings were completed by one observer (RD). Secondly, a fully-automated algorithm was developed to estimate breast volume for each participant using both her T1-w and T2-w images (VaT12). The algorithm included segmenting the breast from the foreground, locating the nipple and mid-sternum positions, coronal profile extraction (based on the protocol for manual segmentation using Dixon images) and pectoral muscle boundary definition. For the fat-water segmentation, a modified version of the automated Van Leemput intensity model and spatial regularization scheme (2) was developed for T2-w images.

A comparison of MRI breast measures obtained from the Dixon and T1-w/T2-w images was conducted in 200 randomly selected women. The distribution of MRI percent water in the Dixon and T1w-/T2-w images were comparable, with similar means and medians (Figure 2). There was a high level of agreement between quintiles of total breast volume across the Dixon and T1-w/T2-w methods, with 76% of women being assigned to the same quintile, and 100% to the same±1 quintile. Agreement between percent water quintiles was more moderate, with 32% of women being assigned to the same quintile, but 89% to the same±1 quintile. However, across methods, breast measures were highly correlated with inter-class correlations ≥0.97, P<0.0001 for both total volume and percent water.

The T1-w/T2-w method was applied to all participants and used in the present analysis as its segmentation process, in contrast to the Dixon approach, is fully-automated, and hence less labour-intensive, and more objective (i.e. observer-independent).

**Figure 1: Axial, sagittal and coronal views of each type of MRI image**

A: Dixon method images, from left to right: axial, sagittal and coronal views.

| 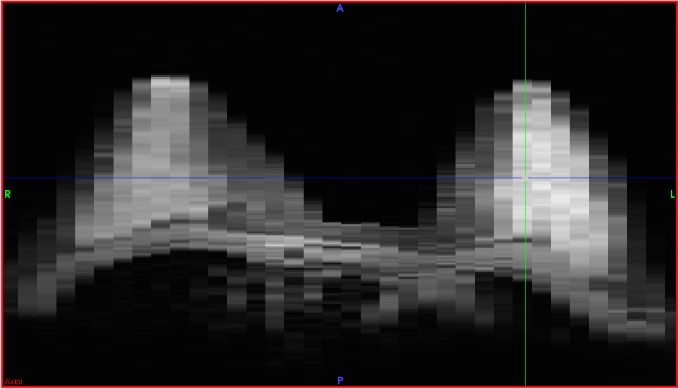 | 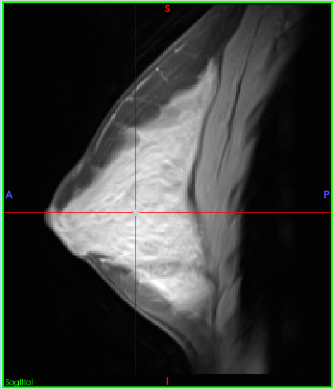 | 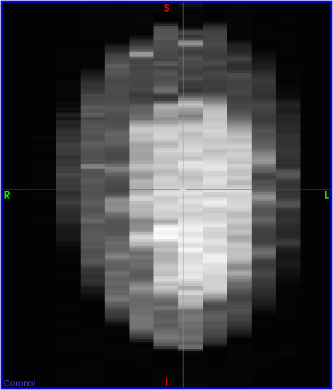 |
| --- | --- | --- |

B: T1-weighted VIBE (T1-w) images, from left to right: axial, sagittal and coronal views.

| 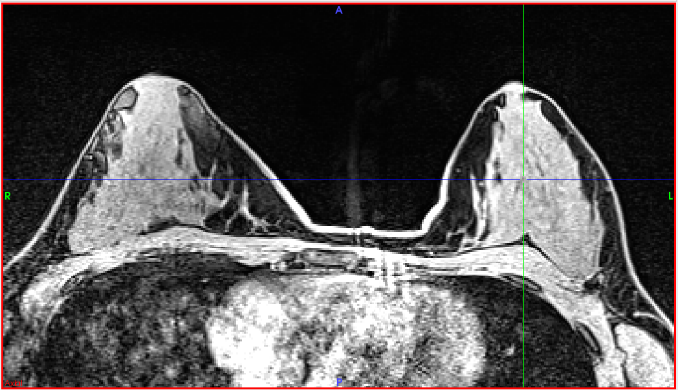 | 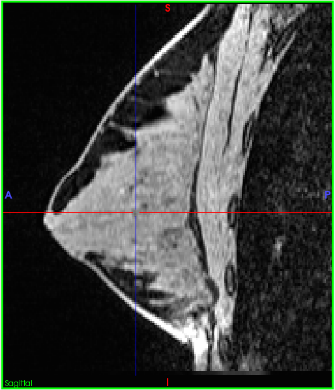 | 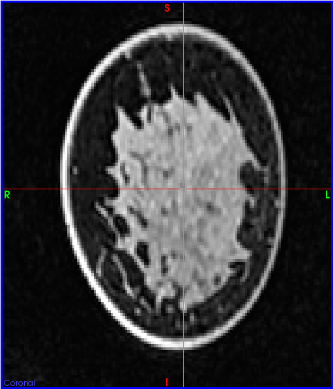 |
| --- | --- | --- |

C: T2-weighted trans-axial (T2-W) images, from left to right: axial, sagittal and coronal views.

| 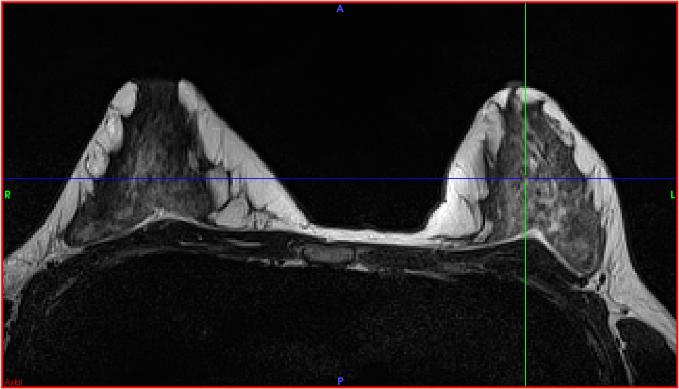 | 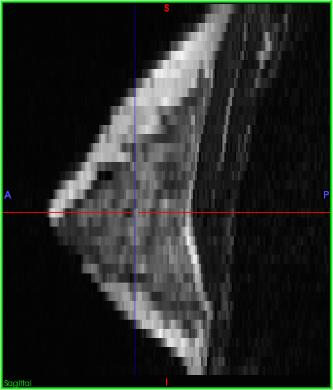 | 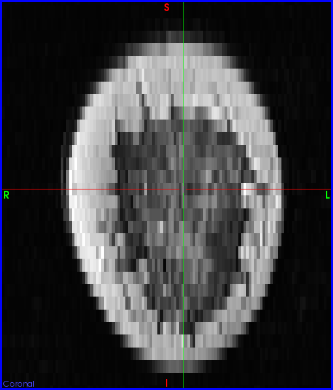 |
| --- | --- | --- |

In the Dixon (A) and T1-w (B) images, the bright, white region represents water, and the dark, black region represents fat (or non-water). In the T2-w (C) images, the dark, black region represents water content, and the bright, white region fat.

**Figure 2: Histograms of the distribution of total breast, fat and water volume estimates (in cm^3^), and of percent water (%), per breast by type of MRI image and segmentation method (n=200)**


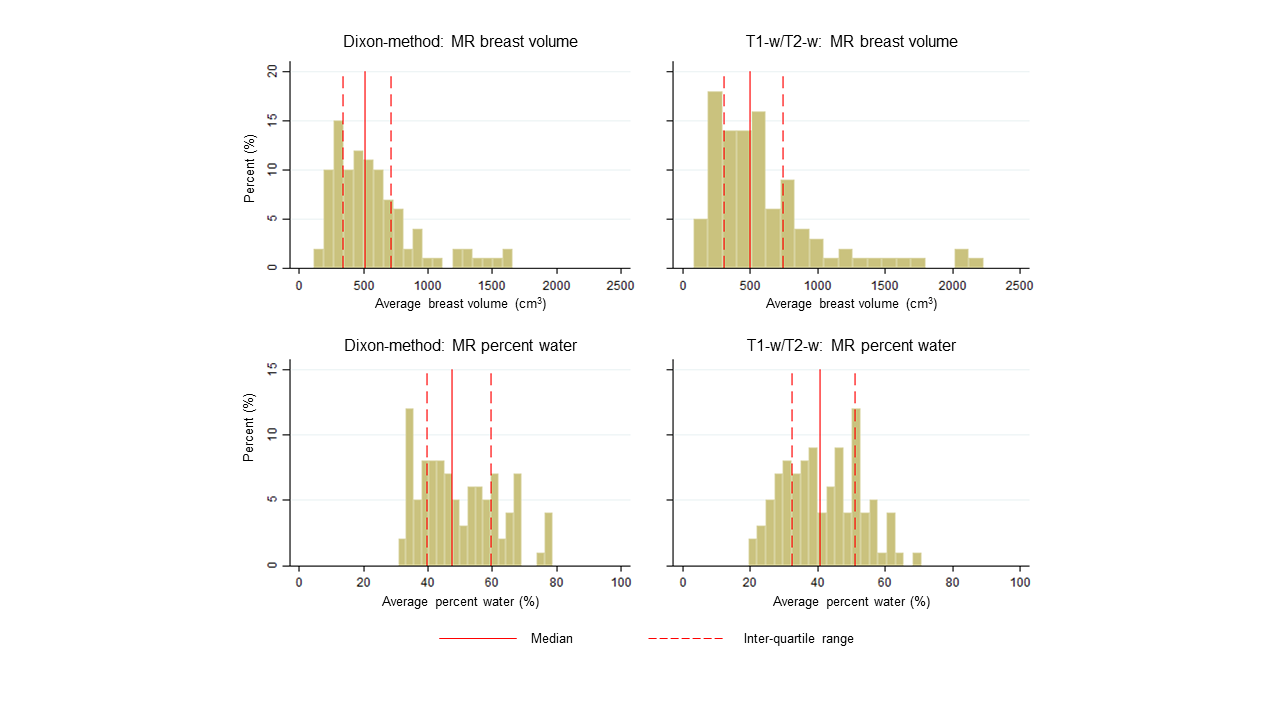


## References

1. Denholm R, Hipwell HJ, Doran JS, et al. Comparison of breast density measures using manual and automated segmentation of three-dimensional Magnetic Resonance images. 2016 [In Review]
2. Van Leemput K, Maes F, Vandermeulen D, Suetens P. Automated model-based tissue classification of MR images of the brain. *IEEE Trans Med Imag* 1999; 18 (10): 897-908.

**Supporting Methods 2: Protocol of the Systematic Review on pre-natal exposures and breast-tissue composition**

(Final version, May 2015)

**Title: Pre-natal exposures and breast-tissue composition**

**Review team:**

Rachel Denholm and Isabel dos Santos Silva

**Background:**

Breast-tissue composition is a strong and independent biomarker of susceptibility to breast cancer (BC), which may, like BC risk itself, be influenced by events early in life when susceptibility to carcinogens is greatest.

**Objectives:**

The main objective is to systematically review published data on associations between maternal, *in-utero* and birth size variables and breast-tissue composition.

Specific aims are:

1. To examine associations between maternal, in utero and birth size variables and breast-tissue composition measures;
2. To identify sources of heterogeneity in study-specific estimates.

**Search strategy:**

We plan to identify and review all published peer-reviewed studies that meet the eligibility criteria described below.

*Eligibility criteria*

Studies will be eligible if (all conditions need to be met):

- Setting: Caucasian populations
- Type of studies: original reports (primary data collection) including cohort and cross-sectional studies. Case-control studies on breast cancer will also be eligible if restricted to controls only
- Date: article published between 1^st^ January 1970 and 25^th^ September 2015
- Language: any language
- Size: no restrictions will be imposed

*Exclusion criteria:*

Studies will be excluded if they focus on:

- non-humans
- males
- non-Caucasian women

Studies will also be excluded if reviews, conference abstracts and proceedings, and general discussion papers

*Search databases:*

The following electronic databases will be searched:

- Pubmed

The results and dates of each search will be recorded.

*Search terms:*

The search will be conducted using specific keywords to identify relevant papers.

*Hand searches:*

Reference lists of all included studies will be cross-checked to identify other potentially relevant studies. In addition, reference lists of reviews, conference papers and discussions articles – which will be ineligible for the review - will be searched and cross-checked.

**Title and abstract screening**

Literature searches of the electronic databases listed above will be conducted and the resulting citations will be downloaded to EndNote software, where duplicate citations will be removed. Any additional citations identified through hand-searches will be added to this database.

The titles and abstracts from this initial database will be screened by one reviewer and classified using the eligibility and exclusion criteria described above as:

- Yes, full paper to be retrieved and screened
- No, exclude
- Unclear

A sample of 10% of the abstracts will also be independently screened by a second reviewer. The reason for exclusion of papers from the review will be documented.

**Full-text screening**

The full-text article for all references classified as “Yes” or “Unclear” from the abstract screen will be retrieved and screened by one author to confirm reporting on the exposures and outcome of interest. Any exclusion of articles from the review will be documented.

**Data abstraction**

A standardised data extraction form will be developed and pre-tested. Any ambiguities will be discussed and the form amended accordingly.

Data will be extracted on the following variables:

- Study identifiers: ID, author(s), year of publication
- Characteristics of the study population: country, study design (e.g. cohort, population-based), study period, eligibility criteria, recruitment and participation rates, and final sample size;
- Pre-natal exposures: maternal age and parity at the time of birth of the participant; maternal height; maternal pre-pregnancy weight and gestational weight gain; maternal smoking and alcohol intake during pregnancy; maternal contraception use; participant’s birth size measures (i.e. weight, length, ponderal index and head circumference at birth) and gestational age; and placenta weight.
- Source/timing of collection of data on the pre-natal exposures: self-reports in adult life; parental reports when the participants were adults; parental report when the participants were children; parental report close to the time of birth of the participant; data extracted from hospital/obstetric records.
- Breast-tissue composition assessment: type of imaging method used (e.g. mammography, dual X-ray absorptiometry (DXA), magnetic resonance imaging (MRI)); type of method used to quantify density (e.g. visual inspection, semi-automated, automated); scale used (e.g. binary, categorical or continuous).
- Socio-demographic characteristics of the participants at breast-tissue composition assessment: age, socio-economic characteristics;
- Reproductive-related variables of the participants: age at menarche; age at first birth; parity, menopausal status, use of oral contraceptives and hormone therapy at the time of breast-tissue composition assessment.

If there are multiple eligible papers from the same study only the one based on the largest sample size, or the one with the most comprehensive exposure data, will be selected for inclusion in the systematic review.

Relevant data from each eligible study will be extracted independently by two reviewers. Each of them will complete the standardised data extraction form. The two resulting databases will be compared to identify discrepancies - these will be discussed and resolved by consensus.

**Study quality assessment**

The two reviewers will independently use the data extracted from each study to assess their quality using a specifically-developed standardised quality assessment form. This assessment form will be developed to capture three domains:

- Potential for selection bias (e.g. study design; participation rates; percentage of the study population with both pre-natal and breast density data);
- Potential for exposure and outcome measurement errors (e.g. source and timing of collection of data on pre-natal variables; method of breast density method used);
- Availability of data on key variables (e.g. age and BMI at time of breast density assessment)

A list of items for each one of the three domains will be developed. For each item, papers will be allocated a score ranging from 0 (if it does not meet the criteria or if the information provided is unclear) to a maximum to be defined (e.g. 1, 4 or 8, depending on the specific item). The overall quality of the study will be expressed as the sum of its item-specific scores. The higher the score the higher the methodological quality of the study, that is the lower the risk that its findings may have been affected by bias.

**Data analysis**

The extracted data will be analysed in STATA (Statistical Software version 14 (StataCorp, Texas).

Basic descriptive analyses will be conducted to summarise information about the study population (e.g. by country, type of study), source and timing of pre-natal variables collected, method used to assess breast density, etc.

Analyses will be conducted separately for each pre-natal exposure. If appropriate, depending on the number and characteristics of the studies included in the review, and on the data reported, pooled effect estimates of the association between a given pre-natal exposure and density breast-tissue composition measure will be estimated using random effects models.

To examine potential sources of heterogeneity, study-specific estimates will be stratified according to relevant factors (e.g. age or menopausal status at mammography) and methodologically relevant variables (e.g. e.g. source of pre-natal exposure data; method used to assess breast-tissue composition; study quality score).

Between-study heterogeneity will be formally assessed using I^2^ (1). The findings will be tabulated and/or displayed graphically using forest plots.

Small study bias will be assessed via funnel plots and the Egger funnel plot asymmetry test (2).

**References**

1. Higgins JP, Thompson SG, Deeks JJ, Altman DG. Measuring inconsistency in meta-analyses. *Br Med J* 2003; 327: 557-60.

2. Egger M, Davey Smith G, Schneider M, Minder C (1997) Bias in meta-analysis detected by a simple, graphical test. *Br Med J* 1997; 315: 629-34.

**Supporting Methods 3: Systematic literature review of maternal, *in-utero* and birth size variables and breast-tissue composition**

**Search terms used in PubMed**

(“mammographic density” OR “mammography” OR “breast density” OR “mammographic” OR “parenchymal patterns”)

**OR**

Mammography (MeSH Term)

**AND**

(“birth size” OR prenatal OR perinatal OR *in-utero* OR “maternal health” OR “birth weight” OR “head circumference” OR “gestational age” OR “birth length” OR “ponderal index”)

**OR**

(“gestational weight gain” OR “placenta” OR (“weight gain” AND pregnancy) OR “post-partum weight”)

**OR**

(maternal **AND** (height OR smoking OR BMI OR weight OR alcohol OR parity OR “age of first birth” OR reproductive OR menarche OR contraception OR (‘mammographic density’ OR “mammography” OR “breast density” OR “mammographic” OR “parenchymal patterns”))

**OR**

birth weight (MeSH Term)

**OR**

maternal age (MeSH Term)

Limits: Humans, 1970-Current

**Data extraction**

Data extraction from all eligible articles was performed independently by two of the authors (RD and IdSS) using a specifically developed standardised data extraction form (Text S2). For each eligible paper data were extracted on country, study years, study design, study population, sample size; average age (mean (SD) or median (IQR)) at the time of breast-tissue composition assessment, method of breast-tissue composition assessment, source and type of pre-natal exposures investigated. Data were also extracted on other relevant covariates such as age, BMI, menopausal status, and use of oral contraceptives (OC) and hormone therapy (HT) at the time of breast-tissue composition assessment. If there was more than one point estimate for the association between a given pre-natal exposure and a breast-tissue composition measure the most adjusted one was chosen for the meta-analysis. Disagreements between authors were discussed and a consensus reached.

**Study quality assessment**

The quality of the papers included in the review was assessed by developing a standardized quality assessment form based on an approach similar to that used by the Cochrane Collaboration. We scored individual parameters based on three broad categories which were chosen to reflect the potential for (i) selection bias (4 parameters) and (ii) measurement errors (5 parameters) and (iii) the availability of data on potential confounders (6 parameters), as indicated below.

Minimizing selection bias

1. *Study Design*

Score 0 if unclear

Score 1 if opportunistic cross-sectional/case-control study

Score 2 if population-based cross-sectional/case-control study

Score 3 if cohort study (or case-control study/case-cohort study nested within a cohort study)

1. *Participation rate*

Score 0 if unclear

Score 1 if <70% of those eligible

Score 2 if ≥70% of those eligible

1. *Percentage of the study population with data on the pre-natal exposures of interest*

Score 0 if unclear

Score 1 if <70% of study population

Score 2 if ≥70% of study population

1. *Percentage of the study population with breast-tissue composition measures*

Score 0 if unclear

Score 1 if <70% of study population

Score 2 if ≥70% of study population

Minimizing exposure and outcome measurement errors

1. *Source / timing of collection of data on the pre-natal exposure variables of interest*

Score 0 if unclear

Score 1 if self-reports in adult life (retrospective)

Score 2 if parental report in adult life of the participants

Score 4 if parental report during childhood of the participants

Score 6 if parental report close to the time of birth of the participants

Score 8 if data extracted from hospital/obstetric records (i.e. prospective)

1. *Type of unit in which the pre-natal variable was collected*

Score 0 if unclear

Score 1 if binary

Score 2 if categorical

Score 4 if quantitative

1. *Type of breast images acquired*

Score 0 if unclear

Score 1 if copies of analogue films

Score 2 if original analogue films

Score 3 if digital mammographic, magnetic resonance imaging, or dual X-ray absorptiometry images (i.e. images do not require digitisation)

1. *Method of breast-tissue composition assessment*

Score 0 if unclear

Score 1 if subjective (e.g. Wolfe, BI-RADS, Cumulus) and not blind to the women’s characteristics (or if not known if blind)

Score 2 if subjective but blind

Score 4 if objective (e.g. fully-automated)

1. *Type of scale used in the breast-tissue composition measurements*

Score 0 if unclear

Score 1 if binary

Score 4 if categorical (more than 2 - e.g. full Wolfe, BI-RADS, 6-category)

Score 8 continuous (e.g. Cumulus, ImageJ-based method)

Minimizing confounding

1. *Age at the time of breast-tissue composition assessment (e.g. age at mammography)*

Score 0 if not adjusted

Score 8 if adjusted

1. *Body mass index (BMI) at the time of breast-tissue composition assessment*

Score 0 if not adjusted

Score 4 if BMI measured >5yrs from breast-tissue composition assessment or if weight was used as a proxy for BMI

Score 8 if adjusted for BMI close to time of breast-tissue composition assessment

1. *How was BMI assessed?*

Score 0 if not reported

Score 1 if self-reported

Score 2 if measured

1. *Menopausal status*

*Sc*ore 0 if not adjusted

Score 2 if adjusted

1. *OC / HT use at the time of breast-tissue composition assessment*

Score 0 if not adjusted

Score 2 if adjusted

1. *Other reproductive-related variables (e.g. menstrual phase, parity)*

Score 0 if not adjusted

Score 1 if adjusted

More weights were given to the quality of the pre-natal exposure and breast-tissue composition data, and adjustment for at least age and BMI at the time of breast-tissue composition assessment. For each parameter, papers were assigned a score ranging as listed above. The overall quality of the study was expressed as the sum of its parameter-specific scores, with possible scores ranging from 0 (lowest) to 59 (highest); the higher the score the higher the methodological quality of the study and, hence, the lower the probability that its findings might have been affected by bias.
