# Supplementary material for: Pre-natal exposures and breast tissue composition: findings from a British pre-birth cohort of young women and a systematic review
Source: Breast Cancer Res. 2016 Oct 12;18:102. doi: 10.1186/s13058-016-0751-z (PMC5059986; doi:10.1186/s13058-016-0751-z)
Supplement: Additional file 2: Table S1. — Mutually adjusted associations of MRI breast tissue measurements in daughters, and mammographic breast measurements in mothers, with age, anthropometry and hormone status at the time of the breast examination. Table S2. Minimally adjusted associations of MRI breast percent water in relation to maternal, in utero and birth size characteristics using complete and imputed data (n = 491), and Dixon-based MRI breast percent water. Table S3. Systematic review of studies investigating the association between birth size measurements, gestational age and percent breast density. Table S4. Systematic review of studies investigating the association between maternal and in utero exposures and percent breast density. (DOCX 103 kb) [file 13058_2016_751_MOESM2_ESM.docx]

**Table S1: Mutually-adjusted associations of MRI breast-tissue measurements in daughters, and mammographic breast measurements in mothers, with age, anthropometry and hormone status at the time of the breast examination**

|  | | | **Relative change in geometric means (95% CI)** | | | |
| --- | --- | --- | --- | --- | --- | --- |
|  | | | **Total breast volume**  **(cm^3^) ^a^** | **Total fat**  **volume**  **(cm^3^) ^a^** | **Total water volume**  **(cm^3^) ^a^** | **Percent water ^a^** |
| **All participating daughters (n=483) ^b^** | | |  |  |  |  |
| Age at MRI (per 1 SD: 11 months) | | | 1.00 (0.99, 1.00) | 1.00 (0.99, 1.00) | 0.99 (0.99, 1.00) | 1.00 (1.00, 1.00) |
| BMI at MRI (per 1 SD: 4.3 kg/m^2^) | | | **1.65 (1.58, 1.71)** | **1.86 (1.78, 1.95)** | **1.36 (1.31, 1.41)** | **0.83 (0.81, 0.84)** |
| Menstrual phase at MRI | Follicular | | 1 (ref) | 1 (ref) | 1 (ref) | 1 (ref) |
|  | Luteal | | 1.05 (0.90, 1.24) | 1.06 (0.89, 1.27) | 1.05 (0.89, 1.23) | 1.00 (0.93, 1.06) |
|  | Irregular period | | 1.05 (0.94, 1.18) | 1.06 (0·93, 1.20) | 1.06 (0.94, 1.18) | 1.00 (0.96, 1.05) |
|  | Hormone contraceptive | | 0.98 (0.81, 1.19) | 1.02 (0·82, 1.26) | 0.92 (0.76, 1.12) | 0.94 (0.87, 1.02) |
|  | | | **Breast area**  **(cm^2^)** | **Non-dense area**  **(cm^2^)** | **Dense area**  **(cm^2^)** | **Percent density** |
| **Mothers with mammographic measurements (n=164) ^c^** | | |  |  |  |  |
| Age at mammogram (per 1 SD: 3.9 yrs) | | | 1.03 (0.97, 1.10) | **0.79 (0.72, 0.87)** | **0.82 (0.75, 0.89)** | **0.79 (0.72, 0.87)** |
| BMI at mammogram (per 1 SD: 4.7 kg/m^2^) | | | **1.35 (1.28, 1.43)** | **0.72 (0.66, 0.78)** | 0.97 (0.89, 1.05) | **0.72 (0.66, 0.78)** |
| Parity at mammogram (per 1 SD: 0.86 children) | | | 0.97 (0.92, 1.03) | 1.05 (0.97, 1.14) | 1.02 (0.95, 1.11) | 1.05 (0.97, 1.14) |
| Menopausal status | | No | 1 (ref) | 1 (ref) | 1 (ref) | 1 (ref) |
|  | | Yes | 0.93 (0.78, 1.11) | 0.96 (0.73, 1.26) | 0.89 (0.69, 1.14) | 0.96 (0.73, 1.26) |

MRI: magnetic resonance imaging; CI: confidence intervals; BMI: body mass index; ref: reference category

^a^ MRI and mammographic breast-tissue measurements were log transformed for the analysis and exponentiated estimated regression parameters, with 95% CI calculated by exponentiating the original 95% CIs are presented. All daughters’ and mothers’ variables were included simultaneously in their respective models.

^b^ Data collected at the time of the MRI examination through the administration of a short questionnaire and measurements of height and weight. See Table 1, footnote (a).

^c^ Data collected at the clinical assessment or self-administered questionnaire conducted closest to the time of mammography (median time interval: 3 years; IQR: 1.5 years).

**Table S2: Minimally-adjusted associations of MRI breast water percent in relation to maternal, *in-utero*, and birth size characteristics using complete and imputed data (n=491), and Dixon-based MRI breast water percent**

|  | | **Relative change in MRI breast water percent geometric means (95% CI) a** | | | | | |
| --- | --- | --- | --- | --- | --- | --- | --- |
|  |  | **Complete data** | | **Imputed data (n=491)** | | **Dixon-based MRI breast water percent (n=199)** | |
|  |  | **n** | **RC (95% CI)** | **%** | **RC (95% CI)** | **n** | **RC (95% CI)** |
| **Maternal characteristics (at participants birth)** | |  |  |  |  |  |  |
| Maternal age of menarche | per 1 SD | 444 | 1.00 (0.98, 1.01) |  | 1.00 (0.98, 1.01) | 191 | 1.01 (0.98, 1.03) |
| Mother ever used contraceptive pill | Yes | 15 | 1 (ref) | 96.2 | 1 (ref) | 194 | 1 (ref) |
|  | No | 439 | 0.99 (0.91, 1.09) | 3.8 | 1.00 (0.91, 1.10) | 2 | 1.07 (0.83, 1.39) |
| Age mother first used contraceptive pill | per 1 SD | 430 | 1.00 (0.98, 1.01) |  | 1.00 (0.98, 1.02) | 192 | 1.00 (0.98, 1.03) |
| Mothers height | per 1 SD | 441 | **1.02 (1.01, 1.04)** |  | **1.03 (1.01, 1.05)** | 194 | 1.01 (0.99, 1.04) |
| Maternal age of first birth | per 1 SD | 458 | 0.99 (0.97, 1.01) |  | 0.99 (0.97, 1.01) | 199 | 1.00 (0.97, 1.02) |
| Mother age at participants birth | per 1 SD | 462 | 1.00 (0.98, 1.01) |  | 1.00 (0.98, 1.01) |  |  |
| Mothers parity at participants birth | 0 | 225 | 1 (ref) | 48.3 | 1 (ref) | 93 | 1 (ref) |
|  | 1 | 162 | 1.03 (0.99, 1.06) | 35.2 | 1.03 (0.99, 1.06) | 70 | 1.03 (0.97, 1.09) |
|  | 2+ | 75 | 1.02 (0.97, 1.06) | 16.6 | 1.01 (0.97, 1.06) | 34 | 1.01 (0.94, 1.08) |
| Mother pre-pregnancy weight (BMI) | per 1 SD | 425 | 1.00 (0.99, 1.02) |  | 1.01 (0.99, 1.02) | 187 | 1.02 (0.99, 1.04) |
|  | Under | 13 | 1.05 (0.95, 1.16) | 3.67 | 1.05 (0.95, 1.17) | 4 | 0.93 (0.77, 1.11) |
|  | Healthy | 351 | 1 (ref) | 80.02 | 1 (ref) | 159 | 1 (ref) |
|  | Over | 67 | 1.03 (0.98, 1.08) | 16.31 | 1.03 (0.98, 1.08) | 24 | 1.02 (0.94, 1.11) |
| Maternal mother had breast cancer | No | 314 | 1 (ref) |  | 1 (ref) | 133 | 1 (ref) |
|  | Yes | 42 | 1.00 (0.95, 1.07) | 12.2 | 1.01 (0.96, 1.07) | 21 | 1.03 (0.95, 1.12) |
| Average percent density (%) | Q1 (<14) | 45 | 1 (ref) |  |  | 24 | 1 (ref) |
|  | Q2 (14-) | 45 | 1.04 (0.96, 1.12) |  |  | 23 | 0.97 (0.87, 1.07) |
|  | Q3 (25.2-) | 44 | 1.06 (0.98, 1.15) |  |  | 15 | 0.98 (0.88, 1.10) |
|  | Q4 (33.8-) | 46 | **1.10 (1.02, 1.20)** |  |  | 21 | 1.00 (0.90, 1.11) |
| **In-utero exposures** |  |  |  |  |  |  |  |
| Placenta weight | per 1 SD | 121 | 1.01 (0.98, 1.04) |  | 1.03 (0.99, 1.07) | 52 | 1.00 (0.95, 1.05) |
| Absolute GWG (kg): wk 0 to delivery | per 1 SD | 417 | 1.00 (0.98, 1.02) |  | 1.00 (0.98, 1.02) | 179 | 0.99 (0.96, 1.01) |
| Consumed alcohol during pregnancy | No | 117 | 1 (ref) | 25.8 | 1 (ref) | 58 | 1 (ref) |
|  | Yes | 344 | 1.00 (0.96, 1.03) | 74.2 | 1.00 (0.96, 1.04) | 140 | 1.02 (0.97, 1.08) |
| Smoked tobacco during pregnancy | No | 414 | 1 (ref) | 88.9 | 1 (ref) | 180 | 1 (ref) |
|  | Yes | 52 | 1.00 (0.95, 1.05) | 11.1 | 1.00 (0.95, 1.06) | 19 | 1.00 (0.92, 1.09) |
| **Perinatal characteristics** |  |  |  |  |  |  |  |
| Birthweight (g) | per 1 SD | 455 | **1.03 (1.02, 1.05)** |  | **1.03 (1.02, 1.05)** | 197 | **1.04 (1.01, 1.07)** |
| Birth length (cm) | per 1 SD | 357 | **1.02 (1.00, 1.03)** |  | **1.02 (1.00, 1.04)** | 147 | **1.04 (1.01, 1.07)** |
| Head circumference (cm) | per 1 SD | 365 | **1.02 (1.01, 1.04)** |  | **1.03 (1.01, 1.04)** | 151 | **1.04 (1.01, 1.07)** |
| Ponderal Index (g/cm3) | per 1 SD | 353 | **1.01 (1.00, 1.03)** |  | **1.01 (1.00, 1.03)** | 145 | 1.01 (0.98, 1.04) |
| Gestational age (weeks)b | <39 | 95 | 1 (ref) | 20.4 | 1 (ref) | 45 | 1 (ref) |
|  | 39 | 103 | 1.00 (0.95, 1.05) | 21.5 | 1.00 (0.95, 1.05) | 39 | 1.01 (0.94, 1.10) |
|  | 40 | 131 | 1.01 (0.96, 1.05) | 27.9 | 1.01 (0.96, 1.05) | 55 | 0.99 (0.92, 1.07) |
|  | 41+ | 140 | 1.01 (0.96, 1.05) | 30.3 | 1.01 (0.96, 1.06) | 62 | 0.97 (0.91, 1.04) |

MRI: magnetic resonance imaging; SD: standard deviation; RC: relative percent change; CI: confidence intervals; BMI: body mass index; GWG: gestational weight gain; Wk: week; ref: reference category

Models adjustments for age, standardised BMI and menstrual phase/ hormone contraceptive use at the time of the MRI examination

^a^ MRI breast water percent measurements were log transformed for the analysis and exponentiated estimated regression parameters, with CI calculated by exponentiating the original 95% CIs are presented.

^b^ Data available only as a categorical variable

**Table S3: Systematic review of studies investigating the association between birth size measures, gestational age, and percent breast density**

| **Author, Country & Study year** | **Sample size, Average age (yrs)** | **MPD assessment method ^a^** | **Source of birth size data** | **Outcome** | **Exposure unit/**  **categories** | **Percent breast density** | | | **Covariates** |
| --- | --- | --- | --- | --- | --- | --- | --- | --- | --- |
|  |  |  |  |  |  | **All women** | **Pre-menopausal** | **Post-menopausal** |  |
| **Birthweight (g)** |  |  |  |  |  |  |  |  |  |
| McCormack, UK 1999 ([33](#_ENREF_33)) | 1,294, 51.5 (SD=1.1) | Wolfe  grade | Hospital records | OR for higher Wolfe grade associated with 1 SD increase | 1 SD | 1.03 (0.92, 1.15) |  |  | **Age**  **Anthropometry**: measured BMI at 53 yrs  **Breast size** |
|  |  |  |  |  |  |  |  |  |  |
| Anderson, Denmark 1991-2001 ([35](#_ENREF_35)) | 8,271,  54.6 (SD=3.4) | Fatty vs· mixed/dense breast ^b^ | Parental reports in childhood | OR fatty vs· mixed/dense | Continuous | OR1:  0.98 (0.90, 1.07)  OR2:  1.11 (1.02, 1.22) |  |  | **Age**  **Other**: birth cohort  For OR2: further adjusted for measured BMI at age 13 yrs |
|  |  |  |  |  |  |  |  |  |  |
| [Pearce, UK 1996-98](#RANGE!_ENREF_6) (50) | 199, 51.5 (IQR: 50.7, 52.0) | Wolfe  grade | Hospital records | OR for higher grade associated with 1 SD increase | z-score for gestational age and sex | 1.32 (1.02, 1.71) |  |  | **Age**  **Anthropometry:** Measured BMI and height at 49-51 yrs  **Reproductive:** age at menarche, age at first pregnancy, menopausal status at mammography, OC/HT use  **Other:** social class at birth and at 49-51 yrs, physical exercise and alcohol intake at 49-51 yrs, cigarette smoking |
| [Lope, Spain 2007-08](#RANGE!_ENREF_8) ([20](#_ENREF_20)) | 3,557,  56  (SD=NK) | Boyd semi-quantitative 6 categories | Self-reports in adulthood | OR for higher category associated with unit increase | Smaller | 0.77 (0.66, 0.98) | 0.58 (0.37, 0.92) | 0.84 (0.63, 1.12) | **Age**  **Anthropometry**: BMI; pre-pubertal height and pre-pubertal weight  **Reproductive:** age at menarche; parity, menopausal status; maternal age at the woman’s birth |
|  |  |  |  |  | Average | 1 (ref) | 1 (ref) | 1 (ref) |  |
|  |  |  |  |  | Larger | 0.94 (0.73, 1.21) | 1.06 (0.63, 1.77) | 0.88 (0.66,1.18) |  |
| [Ekbom, Sweden 1988](#RANGE!_ENREF_10) ([25](#_ENREF_25)) | 370,  <60: 61% | Wolfe  grade | Hospital records | OR for P2/DY vs· N1/P1 | <2500 | 0.97 (0.29, 3.28) |  |  | **Age**  **Breast size**  **Other:** maternal age at woman’s birth; maternal SES; maternal parity, maternal pre-eclampsia or eclampsia· |
|  |  |  |  |  | 2500- | 1 (ref) |  |  |  |
|  |  |  |  |  | 3000- | 1.03 (0.47, 2.27) |  |  |  |
|  |  |  |  |  | 3500- | 1.00 (0.45, 2.22) |  |  |  |
|  |  |  |  |  | 4000+ | 1.39 (0.56, 3.47) |  |  |  |
|  |  |  |  |  | *P trend* | *0*.*53* |  |  |  |
| [Tamimi, Sweden 1993-94](#RANGE!_ENREF_9) ([26](#_ENREF_26)) | 893,  61.2 (SD: 6.8) | Computer assisted | Hospital records | Adjusted OR high (≥50%) vs· low (<50%) categories | <3001 g |  |  | 0.57 (0.18, 1.81) | **Age**  **Anthropometric**: BMI  **Reproductive**: parity; age at menopause |
|  |  |  |  |  | 3001- |  |  | 1 (ref·) |  |
|  |  |  |  |  | 3501- |  |  | 1.68 (0.78, 3.62) |  |
|  |  |  |  |  | >4000 |  |  | 2.91 (1.07, 7.88) |  |
|  |  |  |  |  | *P trend* |  |  | *0.048* |  |
|  |  |  |  |  |  |  |  |  |  |
| Jeffreys, Scotland 2008 ([21](#_ENREF_21)) | 590,  54.1 (range: 40.0, 71.5) | Computer assisted | Self-reports in adulthood | Adjusted OR high (≥50%) vs· low (<50%) categories | <2500 g | 0.27 (0·08, 0.87) | 0.49 (0.06, 3.76) | 0.20 (0.04, 0.97) | **Age** |
|  |  |  |  |  | 2500- | 1.32 (0·61, 2.88) | 0.93 (0.24, 3.68) | 1.55 (0.60, 4.03) |  |
|  |  |  |  |  | 3000- | 1 (ref) | 1 (ref) | 1 (ref) |  |
|  |  |  |  |  | ≥4000 | 0.40 (0.17, 0.92) | 0.20 (0.03, 1.41) | 0.49 (0.19, 1.28) |  |
| [Cerhan, USA 2001](#RANGE!_ENREF_5) ([22](#_ENREF_22)) | 940,  Mean: 60.4 (SD=11.1) | Computer assisted | Self-reports in adulthood | Adjusted mean | <2950 g | 20.1 (19.0, 21.2) | 33.4 (31.1, 35.7) | 17.1 (15.9, 18.3) | **Age**  **Anthropometric**: weight at first follow-up (within 5 yrs from date of mammography for >90% women)  **Reproductive**: age at menarche; age at first birth; parity; menopausal status; OC use; HT use  **Other**: educational level; alcohol use; current smoking status; smoking history |
|  |  |  |  |  | 2950- | 21.0 (20.1, 22.0) | 31.4 (30.0, 32.8) | 19.8 (18.5, 21.0) |  |
|  |  |  |  |  | 3380 | 22.9 (21.8, 24.0) | 35.4 (33.2, 37.5) | 20.2 (18.9, 21.4) |  |
|  |  |  |  |  | ≥3750 | 23.0 (21.8, 24.1) | 34.6 (32.6, 36.6) | 21.0 (19.7, 22.3) |  |
|  |  |  |  |  | *P trend* | *<0.01* | *0.19* | *<0.01* |  |
| [Lokate, The Netherlands 1993-97](#RANGE!_ENREF_7) (51) | 2,588,  50-70 | Computer assisted | Self-reports in adulthood | Adjusted mean PD | <2000 | 22.2 (19.2, 25.4) |  |  | **Age**  **Anthropometric**: BMI, height, leg length  **Reproductive**: age at menarche, age at first birth, parity, menopausal status, OC use, HT use  **Other:** sub-study |
|  |  |  |  |  | 2000- | 21.7 (19.6, 23.9) |  |  |  |
|  |  |  |  |  | 3000- | 21.8 (20.2, 23.4) |  |  |  |
|  |  |  |  |  | 4000+ | 21.0 (18.9, 23.3) |  |  |  |
|  |  |  |  |  | *P trend* | *0.532* |  |  |  |
| **Birth length (cm)** |  |  |  |  |  |  |  |  |  |
| [Ekbom, Sweden 1988](#RANGE!_ENREF_10) ([25](#_ENREF_25)) | 370,  <60: 61% | Wolfe  grade | Hospital records | OR for P2/DY vs· N1/P1 | <49·5 cm | 1 (ref) |  |  | **Age**  **Breast size**  **Other:** maternal age at woman’s birth; maternal SES; maternal parity, maternal pre-eclampsia or eclampsia· |
|  |  |  |  |  | 49·5- | 1.00 (0.52, 1.93) |  |  |  |
|  |  |  |  |  | 51·0- | 0.86 (0.46, 1.63) |  |  |  |
|  |  |  |  |  | ≥52·5 | 1.37 (0.70, 2.68) |  |  |  |
|  |  |  |  |  | *P trend* | *0.52* |  |  |  |
| [Tamimi, Sweden 1993-94](#RANGE!_ENREF_9) ([26](#_ENREF_26)) | 893, 61.2 (SD: 6·8) | Computer assisted | Hospital records | Adjusted OR high (≥50%) vs· low (<50%) categories | <50 |  |  | 0.77 (0.27, 2.21) | **Age**  **Anthropometric**: BMI  **Reproductive**: parity; age at menopause |
|  |  |  |  |  | 50- |  |  | 0.85 (0.29, 2.52) |  |
|  |  |  |  |  | 51- |  |  | 1 (ref) |  |
|  |  |  |  |  | 52- |  |  | 1.10 (0.37, 3.26) |  |
|  |  |  |  |  | ≥53 |  |  | 1.04 (0.35, 3.11) |  |
|  |  |  |  |  | *P trend* |  |  | *0.49* |  |
| **Head circumference (cm)** | |  |  |  |  |  |  |  |  |
| [Tamimi, Sweden 1993-94](#RANGE!_ENREF_9) ([26](#_ENREF_26)) | 893, 61.2 (SD: 6·8) | Computer assisted | Hospital records | Adjusted OR high (≥50%) vs· low (<50%) categories | <34 cm |  |  | 0.66 (0.23, 1.87) | **Age**  **Anthropometric**: BMI  **Reproductive**: parity; age at menopause |
|  |  |  |  |  | 34- |  |  | 0.90 (0.33, 2.44) |  |
|  |  |  |  |  | 35- |  |  | 1 (ref) |  |
|  |  |  |  |  | ≥36 |  |  | 1.72 (0.68, 4.35) |  |
|  |  |  |  |  | *P trend* |  |  | *0.04* |  |
|  |  | |  |  |  |  |  |  |  |
| **Gestational age** |  | |  |  |  |  |  |  |  |
| Pearce, UK 1996-98 (50) | 199, 51.5 (IQR: 50.7, 52.0) | Wolfe grade | Hospital records | OR for higher grade associated with 1 SD increase | Continuous | 0.97 (0.79, 1.19) |  |  | Unadjusted |
| Lope, Spain 2007-08 ([20](#_ENREF_20)) | 3490  56  (SD=NK) | Boyd semi-quantitative 6 categories | Self-reports in adulthood | OR for higher category associated with unit increase | Not premature | 1 (ref) |  |  | **Age**  **Anthropometry**: BMI  **Reproductive:** parity, menopausal status |
|  |  |  |  |  | Premature | 0.80 (0.58, 1.12) |  |  |  |
| Cerhan, USA 2001 ([22](#_ENREF_22)) | 862,  Mean: 60.4 (SD=11·1) | 940,  Mean: 60.4 (SD=11·1) | Computer assisted | Self-reports in adulthood | Pre-term | 19.2 (17.3, 21.1) | 30.5 (26.9, 34.0) | 16.7 (14.4, 19.0) | **Age**  **Anthropometric**: weight at first follow-up (within 5 yrs from date of mammography for >90% women)  **Reproductive**: age at menarche; age at first birth; parity; menopausal status; OC use; HT use  **Other**: educational level; alcohol use; current smoking status; smoking history |
|  |  |  |  |  | Term | 22.0 (21.4, 22.6) | 33.3 (32.2, 34.4) | 20.2 (19.5, 21.0) |  |
|  |  |  |  |  | Post-term | 23.7 (20.9, 26.5) | 34.7 (30.3, 39.0) | 23.0 (19.2, 26.8) |  |
|  |  |  |  |  | *P trend* | *0.07* | *0.25* | *0.07* |  |
| Lokate, The Netherlands 1993-97 (51) | 1,378, 50-70 | Computer assisted | Self-reports in adulthood | Adjusted mean PD | Pre-term (>2 weeks early) |  |  | 22.3 (19.5, 25.4) | **Age**  **Anthropometric**: BMI, height, leg length  **Reproductive**: age at menarche, age at first birth, parity, menopausal status, OC use, HT use  **Other:** sub-study |
|  |  |  |  |  | Term |  |  | 21.0 (19.4, 22.7) |  |
|  |  |  |  |  | Post-term (>2 weeks late) |  |  | 21.3 (17.9, 25.0) |  |

BMI: body mass index; HT: hormone therapy; MPD: mammographic percent density; OC: oral contraceptives; OR: odds ratio; ref: reference category; SES: socio-economic status; SD: standard deviation

^a^ All studies in the review that examined associations with birth size and gestational age were based on mammographic assessment of breast density performed on analogue films.

^b^ Fatty breast was equivalent to BI-RADS (2008) density code 1 and part of code 2; Mixed/dense breast, equivalent to part of BI-RADS code 2, 3, or 4.

**Table S4: Systematic review of studies investigating the association between maternal and *in-utero* exposures and percent breast density**

| **Author, Country & Study year** | **Sample size & Average age** | **Breast-tissue composition assessment** | **Exposure source** | **Outcome** | **Exposure** | | **Percent breast density** | | | **Covariates** |
| --- | --- | --- | --- | --- | --- | --- | --- | --- | --- | --- |
|  |  |  |  |  |  |  | **All women** | **Pre-menopausal** | **Post-menopausal** |  |
| **Maternal percent density** | | | |  |  |  |  |  |  |  |
| Boyd, USA 2003-07 ([12](#_ENREF_12)) | 306 D-M pairs, D: 20.8 (SD=4.9); M: 50.9 (4.9) | Semi-automated percent water on Dixon MRI images | Computer-assisted mammographic percent density | Relative change in percent water for unit increase | Maternal mammo-graphic percent density |  |  | 0·.17 |  | **Age** (D and M)  Anthropometry (weight and height for D and M)  **Reproductive** (D only): age at menarche, current OC use  **Other** (D only): physical activity |
|  |  |  |  |  |  | *P trend* |  | *<0.0001* |  |  |
| Maskarinec, USA ([24](#_ENREF_24)) | 101 D-M pairs (plus 12 mothers had 2 D), M: 47.7 (SD=4.8)  D: 13.9 (SD=1.7) | Computer assisted on DXA images | Maternal DXA percent density | Regression coefficients for 1 SD increase in exposure | Maternal DXA fibroglan-dular percent (%) |  |  | -0.04 |  | **Age** (D and M)  **Anthropometry:** DXA % total body fat (D and M)  Other (D only): ethnicity and Tanner breast stage |
|  |  |  |  |  |  | *P trend* |  | *0.53* |  |  |
| **Maternal age** | |  |  |  |  |  |  |  |  |  |
| Lope, Spain 2007-08 ([20](#_ENREF_20)) | 3,584,  45-68y | Boyd semi-quantitative 6 scale | Retrospective self-report | OR for higher category associated with unit increase | Maternal age (yrs) | <30 | 1 (ref) | 1 (ref) | 1 (ref) | **Age**  **Anthropometric**: BMI, height, leg length  **Reproductive**: age at menarche, age at first birth, parity, menopausal status, OC use, HT use  **Other:** sub-study |
|  |  |  |  |  |  | 30- | 1.01 (0.87, 1.14) | 1.00 (0.72, 1.38) | 1.00 (0.84, 1.18) |  |
|  |  |  |  |  |  | 35- | 1.05 (0.87, 1.27) | 1.15 (0.80, 1.66) | 1.04 (0.84, 1.29) |  |
|  |  |  |  |  |  | >39 | 1.28 (1.03, 1.60) | 1.20 (0.74, 1.93) | 1.32 (1.03, 1.70) |  |
|  |  |  |  |  |  | 5-year trend | 1.04 (0.99, 1.10) | 1.06 (0.95, 1.18) | 1.04 (0.98, 1.10) |  |
| Ekbom, Sweden 1988 (25) | 370,  <60: 61% | Wolfe  grade | Hospital records | OR for P2/DY vs· N1/P1 | Continuous maternal age (yrs) | Per 1-year increment | 0.98 (0.94, 1.02) |  |  | **Age**  **Breast size**  **Other:** maternal age at woman’s birth; maternal SES; maternal parity, maternal pre-eclampsia or eclampsia· |
| Cerhan, USA 2001 ([22](#_ENREF_22)) | 1,550  Mean: 60.4 (SD=11.1) | 940,  Mean: 60.4 (SD=11·1) | Computer assisted | Self-reports in adulthood | Maternal age (yrs) | <19 | 20.8 (19.1, 22.5) | 32·5 (28·8, 36·2) | 18.9 (17.1, 20.6) | **Age**  **Anthropometric**: weight at first follow-up (within 5yrs from date of mammography for >90% women)  **Reproductive**: age at menarche; age at first birth; parity; menopausal status; OC use; HT use;  **Other**: educational level; alcohol use; current smoking status; smoking history |
|  |  |  |  |  |  | 20- | 20.6 (19.8, 21.4) | 31·8 (30·1, 33·4) | 19.0 (18.0, 19.9) |  |
|  |  |  |  |  |  | 25- | 22.2 (21.4, 23.1) | 33·6 (32·0, 35·3) | 20.5 (19.5, 21.5) |  |
|  |  |  |  |  |  | 30- | 20.7 (19.8, 21.6) | 31·9 (29·9, 33·8) | 19.0 (18.0, 19.9) |  |
|  |  |  |  |  |  | ≥35 | 21.6 (20.7, 22.5) | 33·8 (32·0, 35·5) | 19.7 (18.7, 20.7) |  |
|  |  |  |  |  |  | *P trend* | *0.42* | *0.65* | *0.49* |  |
| Lokate, The Netherlands 1993-97 (51) | 2,468, 50-70 | Computer assisted | Self-reports in adulthood | Adjusted mean PD | Continuous maternal age (yrs) | ≤25 |  |  | 20.3 (18.6, 22.0) | **Age**  **Anthropometric**: BMI, height, leg length  **Reproductive**: age at menarche, age at first birth, parity; menopausal status, OC use, HT use  **Other:** sub-study, paternal age |
|  |  |  |  |  |  | 26- |  |  | 20.6 (19.1, 22.2) |  |
|  |  |  |  |  |  | 30- |  |  | 20.7 (19.2, 22.3) |  |
|  |  |  |  |  |  | >33 |  |  | 21.2 (19.5, 22.9) |  |
|  |  |  |  |  |  | *P trend* |  |  | *0.391* |  |
|  | |  |  |  |  |  |  |  |  |  |
| **Maternal parity** | |  |  |  |  |  |  |  |  |  |
| Ekbom, Sweden 1988 ([25](#_ENREF_25)) | 370,  <60: 61% | Wolfe  grade | Hospital records | OR for P2/DY vs· N1/P1 | Maternal parity | 1 | 1 (ref) |  |  | **Age**  **Breast size**  **Other:** maternal age at woman’s birth; maternal SES; maternal parity, maternal pre-eclampsia or eclampsia· |
|  |  |  |  |  |  | ≥2 | 1.24 (0.74, 2.07) |  |  |  |
| Cerhan, USA, 2001 ([22](#_ENREF_22)) | 1636,  Mean: 60.4 (SD=11.1) | Computer assisted | Self-reports in adulthood | Adjusted mean | Birth rank | 1^st^ child | 21.1 (20.3, 21.9) | 32.5 (30.8, 34.2) | 19.3 (18.4, 20.2) | **Age**  **Anthropometric**: weight at first follow-up (within 5yrs from date of mammography for >90% women)  **Reproductive**: age at menarche; age at first birth; parity; menopausal status; OC use; HT use;  **Other**: educational level; alcohol use; current smoking status; smoking history |
|  |  |  |  |  |  | 2^nd^ child | 21.3 (20.4, 22.3) | 35.8 (34.1, 37.5) | 18.7 (17.6, 19.7) |  |
|  |  |  |  |  |  | 3^rd^ child | 21.5 (20.4, 22.5) | 33.3 (31.4, 35.2) | 19.8 (18.6, 21.0) |  |
|  |  |  |  |  |  | 4^th^ child | 22.0 (20.9, 23.1) | 32.4 (30.1, 34.8) | 20.6 (19.4, 21.9) |  |
|  |  |  |  |  |  | >5^th^ child | 20.7 (19.9, 21.6) | 32.3 (30.5, 34.2) | 19.2 (18.3, 20.1) |  |
|  |  |  |  |  |  | *P trend* | *0.91* | *0.39* | *0.39* |  |
| Lokate, The Netherlands 1993-97 ([48](#_ENREF_48)) | 2,527, 50-70 | Computer assisted | Self-reports in adulthood | Adjusted mean PD | Birth rank | Eldest |  |  | 20.7 (19.3, 22.2) | **Age**  **Anthropometric**: BMI, height, leg length  **Reproductive**: age at menarche, age at first birth, parity, menopausal status, OC use, HT use  **Other:** sub-study |
|  |  |  |  |  |  | 2- |  |  | 20.5 (19.2, 21.8) |  |
|  |  |  |  |  |  | 6- |  |  | 20.6 (18.8, 22.4) |  |
|  |  |  |  |  |  | >10 |  |  | 20.2 (16.2, 24.7) |  |
|  |  |  |  |  |  | *P trend* |  |  | *0.740* |  |
|  |  |  |  |  |  | Continuous |  |  | 0.01 (-0.01, 0.03) |  |
| **Prenatal exposure to cigarette smoke** | | | |  |  |  |  |  |  |  |
| Cerhan, USA 2001 ([22](#_ENREF_22)) | 1,553,  Mean: 60.4 (SD=11·1) | Computer assisted | Self-reports in adulthood | Adjusted mean | Mother smoked in pregnancy | No | 21.3 (20.8, 21.7) | 32.6 (31.6, 33.6) | 19.5 (19.1, 20.0) | **Age**  **Anthropometric**: weight at first follow-up (within 5yrs from date of mammography for >90% women)  **Reproductive**: age at menarche; age at first birth; parity; menopausal status; OC use; HT use  **Other**: educational level; alcohol use; current smoking status; smoking history |
|  |  |  |  |  |  | Yes | 20.9 (19.4, 22.3) | 33.2 (31.4, 35.0) | 17.6 (15.5, 19.8) |  |
| Terry, USA ([23](#_ENREF_23)) | 678;  44·1y (SD=2.3) | Computer assisted | Parental reports in *in-utero* | Absolute change for unit increase in exposure | Pre-natal exposure to smoking | No |  | 1 (ref) |  | **Age**  **Anthropometric:** BMI  **Other**: birth weight, birth length, age at menarche, maternal education; adult smoking status |
|  |  |  |  |  |  | Yes |  | -2.72 (-5.68, 0.24) |  |  |
|  |  |  |  |  | Maternal no· packs of cigarettes/ day | None |  | 1 (ref) |  | **Age**  **Anthropometric:** BMI  **Other**: maternal education; adult smoking status |
|  |  |  |  |  |  | 0- |  | -2.05 (-5.11, 1.02) |  |  |
|  |  |  |  |  |  | ½- |  | -2.01 (-5.66, 1.64) |  |  |
|  |  |  |  |  |  | ≥1 pack |  | -3.74 (-7.11, -0.37) |  |  |
|  |  |  |  |  |  | *P trend* |  | *0.02* |  |  |
|  | | |  |  |  |  |  |  |  |  |
| **Prenatal exposure to alcohol** | | |  |  |  |  |  |  |  |  |
| Cerhan, USA 2001 ([22](#_ENREF_22)) | 1,331, Mean: 60.4 (SD=11.1) | Computer assisted | Self-reports in adulthood | Adjusted mean | Mother drank alcohol during pregnancy | No | 21.2 (20.7, 21.6) | 32.6 (31.6, 33.6) | 19.4 (18.9, 19.9) | **Age**  **Anthropometric**: weight at first follow-up (within 5yrs from date of mammography for >90% women)  **Reproductive**: age at menarche; age at first birth; parity; menopausal status; OC use; HT use  **Other**: educational level; alcohol use; current smoking status; smoking history |
|  |  |  |  |  |  | Yes | 21.4 (19.7, 23.2) | 33.1 (30.9, 35.2) | 20.0 (17.4, 22.6) |  |
| **Placental weight** | |  |  |  |  |  |  |  |  |  |
| Ekbom, Sweden 1988 ([25](#_ENREF_25)) | 370,  <60: 61% | Wolfe  grade | Hospital records | OR for P2/DY vs· N1/P1 | Placenta weight (g) | <550 g | 1 (ref) |  |  | **Age**  **Breast size**  **Other:** maternal age at woman’s birth; maternal SES; maternal parity, maternal pre-eclampsia or eclampsia· |
|  |  |  |  |  |  | 550- | 1.67 (0.83, 3.40) |  |  |  |
|  |  |  |  |  |  | 630- | 1.76 (0.89, 3.45) |  |  |  |
|  |  |  |  |  |  | 730+ | 2.34 (1.17, 4.68) |  |  |  |
|  |  |  |  |  |  | *P trend* | *0.02* |  |  |  |

D: daughters; M: Mothers; MPD: mammographic percent density; MRI; magnetic resonance imaging; DXA: dual X-ray absorptiometry; BMI: body mass index; HT: hormone therapy; MD: mammographic density; OC: oral contraceptives; OR: odds ratio; ref·: reference category; SES: socio-economic status; SD: standard deviation
