# Supplementary material for: Pre-natal exposures and breast tissue composition: findings from a British pre-birth cohort of young women and a systematic review
Source: Breast Cancer Res. 2016 Oct 12;18:102. doi: 10.1186/s13058-016-0751-z (PMC5059986; doi:10.1186/s13058-016-0751-z)
Supplement: Additional file 3: Figure S1. — Correlation between participants’ MRI breast tissue measurements and their mothers’ mammographic density measurements (n = 164). Figure S2. Predicted MRI breast percent water geometric means in relation to categories of maternal height, maternal mammographic percent density and participant’s size at birth (minimally adjusted estimates). Figure S3. Preferred Reporting Items for Systematic Reviews and Meta-Analyses (PRISMA) flow diagram of the systematic review. Figure S4. a and b Funnel plots for the meta-analysis of birthweight. a Number studies = 9. b Number studies = 8. (c) Funnel plot for the meta-analysis of maternal age (n = 5 studies) and percent breast density. (DOCX 497 kb) [file 13058_2016_751_MOESM3_ESM.docx]

**Figure S1: Correlation between participants’ MRI breast-tissue measurements and their mothers’ mammographic density measurements (n=164)**


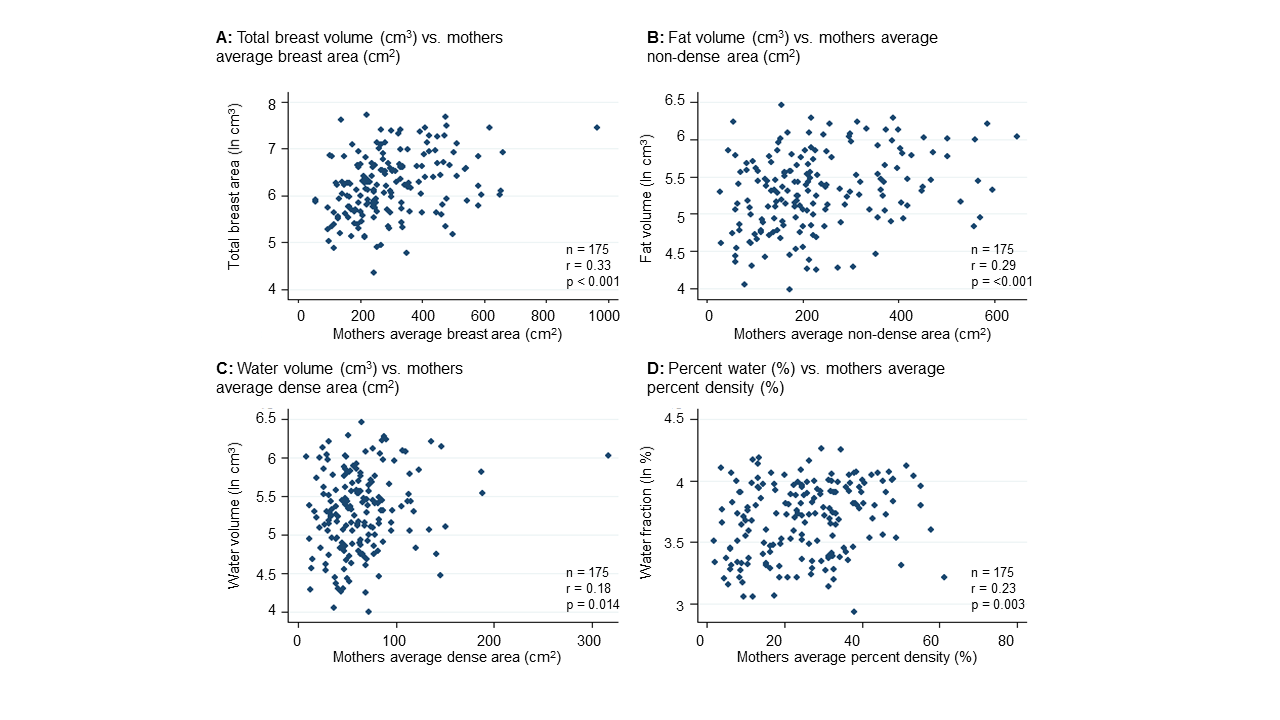


MRI: magnetic resonance imaging; r: Pearson’s correlation coefficient

**Figure S2: Predicted MRI breast percent water geometric means in relation to categories of maternal height, maternal mammographic percent density, and participant’s size at birth (minimally-adjusted estimates)
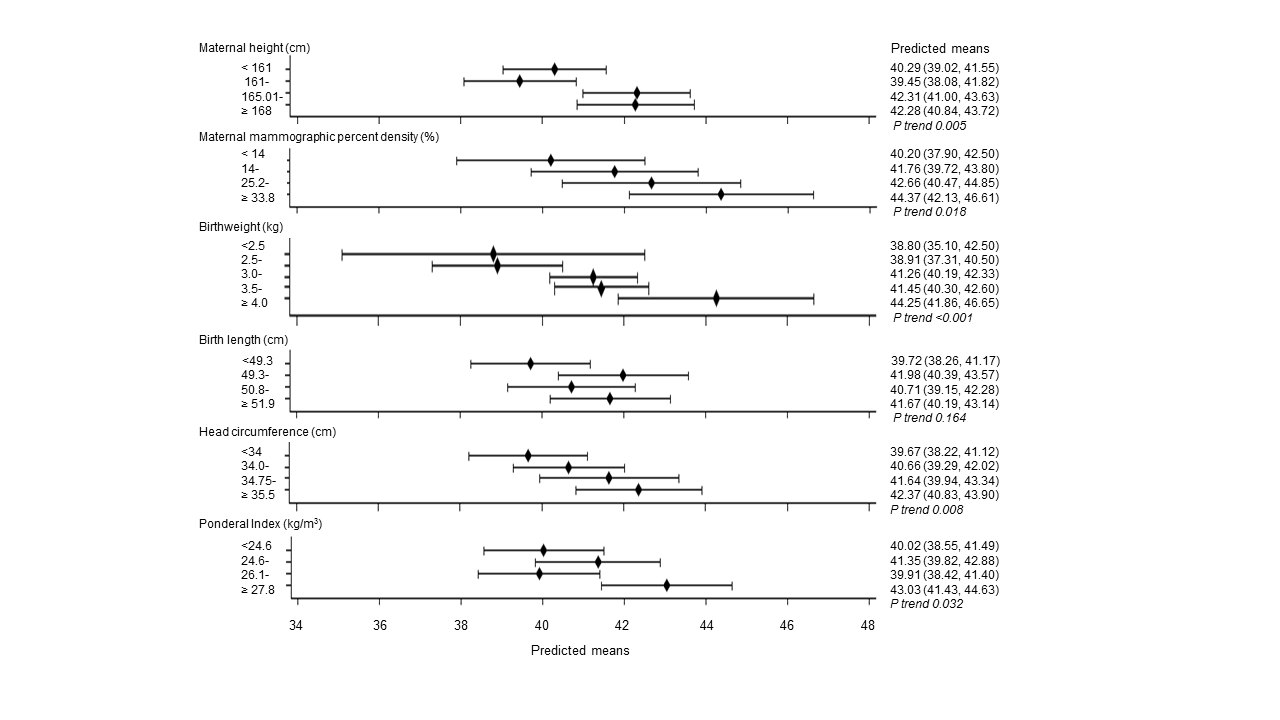
**

MRI: magnetic resonance imaging

MRI breast measures were log transformed, and exponentiated estimated regression parameters, with 95% confidence intervals (CI) calculated by exponentiating the original 95% Cis are presented.

Models adjusted for the participant’s age, BMI and menstrual phase/hormone contraceptive use at MRI and, where appropriate, mother’s age and BMI at mammography. Continuous variables were centred at the mean.

**Figure S3: PRISMA flow diagram of the systematic review**

Full-text articles excluded (n = 18)

Outcome breast cancer (n=9)

Not a relevant exposure measure (n=7)

Review article (n=1)

Non-human study (n=1)

Studies included in quantitative synthesis (meta-analysis)
(n = 9)

Studies included in qualitative synthesis
(n = 12)

Full-text articles assessed for eligibility
(n = 30)

Records excluded
(n = 178)

Records screened
(n = 208)

Records after duplicates removed
(n = 208)

## Identification

## Screening

## Included

## Eligibility

Additional records identified through other sources
(n = 0)

Records identified through database searching
(n = 294)

**Figure S4: Funnel plots for the meta-analysis of birth weight (A: n studies = 9; and B: n=8) and maternal age (C: n=5) and percent breast density**

**
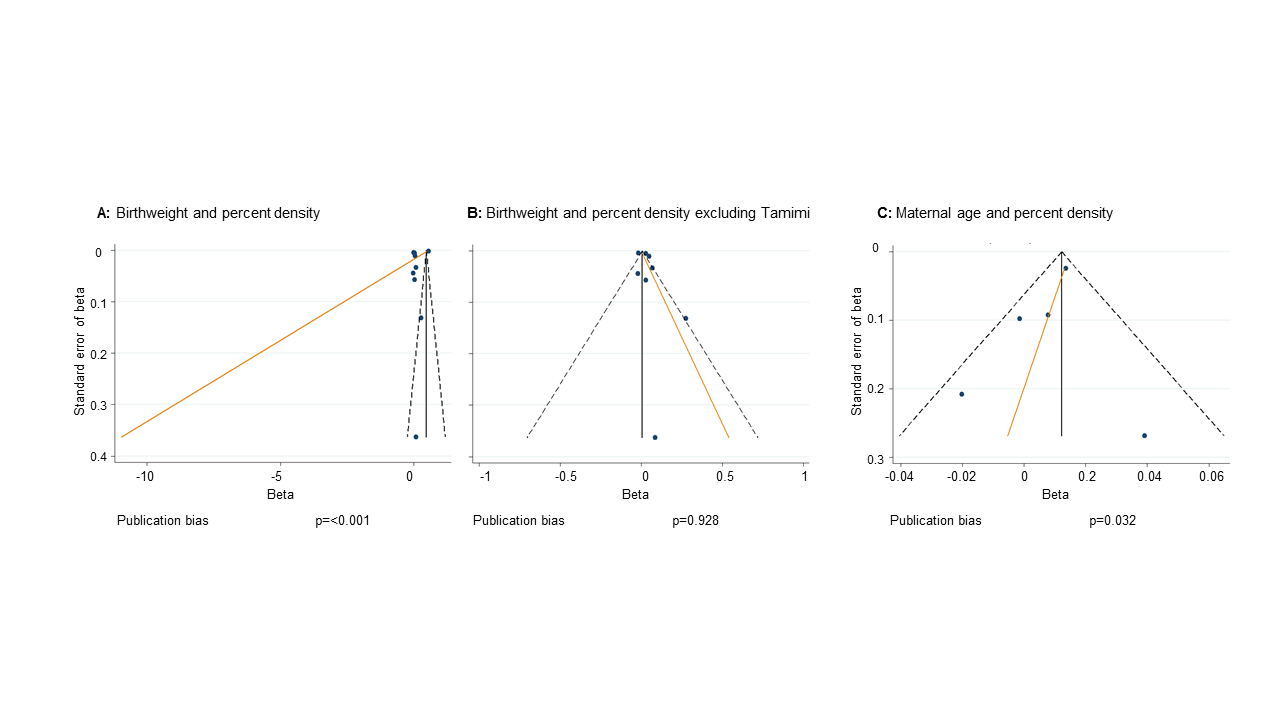
**

The fitted line corresponds to the Egger regression test for funnel-plot asymmetry

Publication bias corresponds to the Egger test, the null hypothesis being that the funnel plot is symmetrical. Evidence against the null hypothesis indicates that there is a linear association between effect size and its standard error, indicating publication bias
